# Pharmacological interrogation of TrkA-mediated mechanisms in hippocampal-dependent memory consolidation

**DOI:** 10.1371/journal.pone.0218036

**Published:** 2019-06-24

**Authors:** Sylvia Josephy-Hernandez, Iulia Pirvulescu, Mario Maira, Tahar Aboulkassim, Tak Pan Wong, R. Anne McKinney, H. Uri Saragovi

**Affiliations:** 1 Lady Davis Institute-Jewish General Hospital, Montreal, Quebec, Canada; 2 Integrated Program in Neuroscience, McGill University, Montreal, Quebec, Canada; 3 Department of Pharmacology and Therapeutics, McGill University, Montreal, Quebec, Canada; 4 Douglas Mental Health University Institute, Montreal, Quebec, Canada; 5 Department of Psychiatry, McGill University, Montreal, Quebec, Canada; Nathan S Kline Institute, UNITED STATES

## Abstract

In the brain, the TrkA receptor for Nerve Growth Factor (NGF) is expressed primarily in the cholinergic system. TrkA/NGF support neuronal health and function, and deficiencies in this axis are associated with progressive cholinergic neuron atrophy and death, and with cognitive deficit in disorders such as Down’s syndrome and Alzheimer’s disease. These observations led to the hypothesis that TrkA agonists may rescue atrophic cholinergic neurons and benefit cognition. Indeed, a small molecule TrkA partial agonist called D3 normalized TrkA signals and improved memory in cognitive impairment models of ageing and an APP mouse model of Alzheimer’s disease. Paradoxically, in young healthy mice chronic delivery of D3 caused impaired memory without impairing learning, a form of anterograde amnesia. Here, we use this as a model to study the mechanisms of impaired memory. In young healthy mice acute or chronic treatment with D3 induces hyperactivation of TrkA-mediated signals in hippocampus, and causes a deficit in hippocampal-dependent memory consolidation proximal to drug exposure, without affecting learning or memory retrieval. The impairment after acute drug exposure is reversible. The impairment after long-term drug exposure is irreversible, likely due to a decrease in hippocampal CA1 neuron basal arborization. These findings support the notion of a homeostatic role for TrkA in memory, and demonstrate the differential outcomes of TrkA (hyper)activation in healthy versus disease states.

## Introduction

Alzheimer’s disease (AD) is the most common type of dementia, but the etiology and pathophysiology remain elusive, and this contributes to the lack of effective treatments [1]. Cholinergic neurons are key to learning and memory, and their atrophy underlies the memory-impairment phenotype of AD and ageing [2]. However, the cholinergic mechanisms that contribute to data processing (learning), data consolidation (storage), data retrieval (recall), and reconsolidation after recall are still poorly understood at a molecular level [3].

TrkA receptors, and its ligand the neurotrophin Nerve Growth Factor (NGF), are central to cholinergic neuron health, phenotype, function, and synaptic plasticity [4–6], and play a key role in normal hippocampal-dependent memory [7]. Alterations to TrkA and NGF have been described as early markers associated with cholinergic neuron atrophy and death in diseases of cognition. For example, degradation or neutralization of NGF [8, 9], impaired NGF transport [10, 11], lower TrkA density [12], or antagonists of TrkA [13] reduce the cholinergic phenotype, and cause memory impairment. Relative to cognitively normal brains, brains from Mild Cognitive Impairment (MCI) patients had low TrkA density and high cholinergic neuron atrophy. The data suggest that phenotypic TrkA loss or silencing *precedes* cholinergic neuronal death [14]. In AD patients the loss of TrkA was even more severe and there was frank cholinergic neuronal death [15]. Similar data were reported for cholinergic neurons in the brains of aged rats with cognitive impairment [12]. These data suggest an inverse relationship between TrkA density/activity and cholinergic atrophy and death, and a direct relationship between TrkA density/activity and cognitive state.

These observations have led to the hypothesis that TrkA agonists may rescue atrophic cholinergic neurons and benefit cognition or memory in disease states [16]. NGF protein has been used as a therapeutic agent to activate TrkA, but has mostly failed due to poor stability and lack of receptor specificity because NGF also binds to p75 receptors leading to activation of unintended pathways [17, 18]. As an alternative we used a small-molecule selective TrkA agonist called D3, which does not bind to p75, to evaluate the specific role of TrkA in memory [19, 20]. *In vitro*, D3 activates TrkA phosphorylation and trophic signals, and potentiates the action of limiting doses of NGF [21]. *In vivo*, D3 improves spatial Long Term Memory (LTM) in memory-impaired aged rats [22], and improves learning and spatial Short Term Memory (STM) in amyloid precursor protein (APP) over-expressing mice [4]. Paradoxically, while in impaired rodents D3 improves memory, in healthy young mice D3 had the opposite effect: it was detrimental to LTM [4] through mechanisms that were not identified.

Here, we address this paradox and show that acute or chronic treatment with D3 in healthy young mice leads to hyperactivation of TrkA signals in the hippocampus, and inhibits hippocampal-dependent memory consolidation of tasks learned proximal to drug exposure. The defect caused by acute drug treatment is reversible, but after chronic drug treatment is irreversible likely due to a decrease in CA1 neuron basal arborization. There were no effects on learning or memory retrieval in either paradigm. These data uncover novel TrkA-dependent mechanisms for the normal consolidation of memory.

## Materials and methods

### Mice

All animal procedures respected the Canadian Association for Laboratory Animal Science guidelines for use of animals in research, and all protocols were approved by the McGill University Animal Care Committees. C57BL/6 wild-type male mice, 4–6 months old (Charles River and Harlan Laboratories) were used for all experiments except for hippocampal branching analysis. A Thy-1 GFP line M mouse (gift of Dr. David Stellwagen, McGill University, Montreal, Canada) was bred with C57BL/6 wild-type females (Jackson Labs) and mixed offspring was used in hippocampal branching analysis experiments. All mice and treatments were randomized, with researchers blinded to all treatments during data collection and quantification.

### Intracerebroventricular (ICV) compound delivery

Vehicle (artificial cerebrospinal fluid–aCSF: 150mM NaCl, 1.8 mM CaCl_2_, 1.2 mM MgSO_4_, 2 mM K_2_HPO_4_, 10mM glucose, 0.001% mouse serum) and D3 (diluted in aCSF) were delivered via two routes: ICV injection for the acute delivery studies, and Alzet osmotic pumps (Cupertino, CA) for the 2-week delivery studies. D3 was manufactured *in house* as we reported [21]. Quality control demonstrated a single HPLC product, of the expected mass and structure (by Mass Spectrometry, and Nuclear Magnetic Resonance).

Acute delivery protocols were adapted from DeVos (2013) [23]. Briefly, mice were anesthetized with isoflurane and fixed in a stereotactic frame (Kopf Instruments, Tujunga, CA). An incision was made at the midline and a small hole drilled at 0.46 mm posteriorly and 1 mm laterally from Bregma. A Hamilton syringe (Reno, NV) connected to a microinjection unit was inserted 2 mm vertically in the brain targeting the lateral ventricle. 5 minutes were allowed for the compound or aCSF to diffuse before extracting the Hamilton syringe. A maximum of 5 μl was injected, containing either 9 μg or 15 μg of D3, or aCSF.

Chronic delivery protocols were adapted from DeVos (2013) [23] and Aboulkassim (2011) [4]. Briefly, Alzet pumps were primed in sterile PBS overnight at 37° C and loaded with 100 μL (40 μg) of D3 or aCSF. The cannula was placed with a holder (Kopf Instruments) at the same coordinates as described above (targeting the right lateral ventricle) and glued with Loctite superglue. The pump was positioned subcutaneously on the back of the mice.

A flowchart of disease progression and acute and chronic treatments with D3 is shown (S1 Fig).

### Biochemical assays

The hippocampus, cortex and nucleus basalis were dissected and analyzed separately. Whole cell lysates were prepared in ice-cold lysis buffer (20 nM Tris HCl pH 7.5, 137 mM NaCl, 2 mM EDTA pH 8, 4% NP40 detergent) with protease and phosphatase inhibitors (ROCHE). After SDS-PAGE and Western Blot transfer, membranes were studied with primary antibodies: pCREB 1:1,500 (Cell Signaling 9198 or Millipore 556003), CREB 1:1,500 (Cell Signaling 9104), PKCδ 1:1,000 (Invitrogen 38–1400), pMAPK 1:2,000 (Cell Signaling 4370), MAPK 1:2,000 (Cell Signaling 4695), pCaMKII 1:1,000 (Cell Signaling 3361), CaMKII 1:3,000 (Cell Signaling 4436), Actin 1:4,000 (SIGMA A2066), pAkt 1:1,000 (Cell Signaling 4060), Akt 1:1,000 (Cell Signaling 9272), pErk5 1:1,000 (Millipore 07–507), Erk5 1:1,000 (Cell Signaling 3372), β-Tubulin-III 1:10,000 (SIGMA T2200); followed by horseradish peroxidase-conjugated secondary antibodies at 1:10,000. Proteins were visualized through enhanced chemiluminescence. Quantification was performed through optical densitometry using the ImageJ free software (http://imagej.nih.gov/ij/). Proteins were standardized to α-actin (chronic delivery group for Erk5, CREB, and Akt) and β-tubulin-III (acute and chronic delivery groups for CREB), while phosphorylated proteins were standardized against their respective total proteins.

Immunoprecipitations were as described [24] using a total-TrkA antibody (Santa Cruz sc-118). After SDS-PAGE and Western Blot transfer, membranes were studied to detect p-TrkA with 4G10 antibody (pan-phosphotyrosine antibody Millipore 16–103, 1:10,000 dilution), and total TrkA in the same membranes using the total-TrkA antibody (Santa Cruz sc-118, 1:1,000 dilution).

### Neuronal branching and spine density analysis

Acute and chronic treatments used Thy-1 GFP line M mice to assess neuronal branching, dendritic spine density and spine morphology [25]. Male mice average 4 months old, and female mice average 5 months old were used. The mice treated with acute ICV injections also were tested by MWM to confirm the behavioral effect of D3; and these mice were sacrificed 2 weeks post-treatment (after the second probe trial of the MWM). The mice that were treated chronically with D3 for 2 weeks were sacrificed immediately after treatment completion. The mice were perfused and the brains were dissected and fixed as described above, the brains were cryo-sectioned into 50 μm thick slices. The slides were then covered with VECTASHIELD antifade mounting medium with DAPI. Confocal images were obtained at a magnification of 63X plus a 5X digital zoom for the spine analyses. The Imaris software (http://www.bitplane.com/) was used to perform the analysis. Filament tracing tools were used to determine total branching points and to perform Sholl analysis, on basal and apical branching, of neurons in the CA1 and CA3 region of the hippocampus. Two cells from the CA1 region and one from the CA3 were quantified per mouse, based on being able to see the entirety of the neuron for proper comparison. Established guidelines [26] were followed to quantify spine density, mean spine length and mean spine volume in tertiary dendrites in the same hippocampal regions.

### Imaging

Epifluorescence images were collected using the Leica DM LB 2 microscope equipped with the LAS acquisition software and a Leica DFC480 camera for detection. Confocal images were collected using a Zeiss (Oberkochen, Germany) Axiovert 200M inverted microscope equipped with the LSM 5 Pascal point laser module, the LSM AIM acquisition software, and 2 PMT detectors for spectral detection. Merging of images, analysis and quantification were performed with the Image J, Volocity, and Imaris softwares.

### Morris Water Maze (MWM)

MWM was as described [4, 27], with a three-day training/acclimatization with a visible platform, and to exclude animals with visual or motor deficits. After these three days, the mice were treated acutely with D3 (9 μg or 15 μg total dose) or aCSF. After a one-day recovery, visual cues were re-arranged and the platform was placed submerged in a new quadrant which remained constant for the next five days (learning period). Each day, escape latencies were recorded from three different starting positions for each mouse, with an inter-trial time not exceeding 45 minutes. On the last day of training, after a 2-hour resting period, the hidden platform was removed and the mice were allowed to swim for 60 s (probe trial 1, Short Term Memory—STM). One week later, the groups were tested on a second probe trial (probe trial 2, Long Term Memory—LTM). The performance in both probe trials was quantified as the percentage of the time the mice spent swimming in the quadrant where the platform had been (target quadrant). These tests, starting with the learning phase, were repeated 2 and 3 months after treatment. HVS Image Software for Morris Water Maze was used to quantify latencies and tracking.

### Statistical analysis

All measures are reported as mean ± SEM. All measurements were assessed for normality using the Shapiro-Wilks test. All comparisons between two groups were performed with unpaired Student’s t-test; all comparisons between three or more groups were performed with between/within analysis of variance (ANOVA) and Newman-Keuls post-hoc pairwise comparison tests. p≤ 0.05 was considered significant.

## Results

Previously, we showed that a chronic 2-week delivery of D3 provided memory benefits in cognitively impaired mice and rats. However, in young healthy mice a chronic 2-week delivery of D3 *caused* memory impairment without causing a learning deficit. This unexpected effect lasted for months after drug wash-off [4, 22], suggesting long-lasting transcriptional or anatomical changes caused by drug treatment in young healthy mice. Here, we explore the mechanisms of memory impairment and compare the biochemical, anatomical, and behavioral effects of acute and chronic D3-treatment paradigms.

### Acute delivery of D3 impairs spatial memory in healthy young mice

In young healthy mice, after *acute* ICV delivery of D3 or vehicle control, mice were trained in the MWM for five days. All mice learned the task at the same rate, indicating that there is no learning impairment after acute treatment with D3.

**Fig 1:**
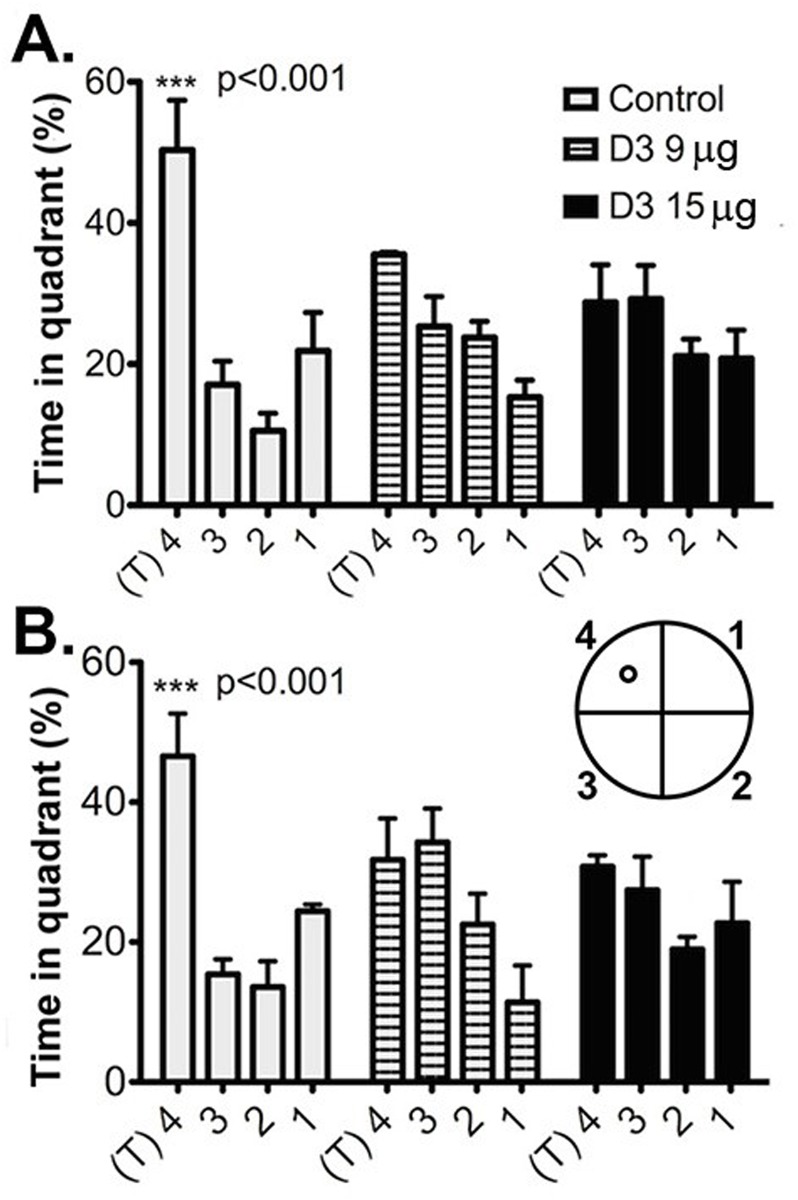
D3-treated mice do not spend significantly more time in the target quadrant during the probe trials. This experiment was replicated two independent times. **(A)** Probe Trial 1: Vehicle-treated (control, n = 4) mice spent significantly more time in the target quadrant ‘4’ (T) than in the remaining quadrants (p<0.001). Neither the D3 9μg-treated mice (n = 3) nor the D3 15 μg-treated mice (n = 5) spent significantly more time in the target quadrant. **(B)** Probe Trial 2: One week later, equivalent results were obtained. Controls spent significantly more time in the target quadrant (p<0.001), mice treated with 9 μg had a significant preference for a quadrant, though it was not the target quadrant (p = 0.01), and mice treated with 15 μg of D3 did not spend significantly more time in any specific quadrant.

In testing for short term memory (STM) 2 hours after the learning phase (probe trial 1) the control group spent significantly more time in the target quadrant whereas the D3-treated groups did not (ANOVA p<10^−5^; df = 51; n = 12). Control untreated mice spent on average 50.3% of the time in the target quadrant. In contrast, the D3 treated mice spent a lower amount of time in the target quadrant, not significantly different from non-target quadrants for the D3 9 μg group (35.5%, p = 0.09) and the D3 15 μg group (28.7%, p = 0.34, df = 51) (Fig 1A). This indicates a STM deficit in D3-treated mice. The impairment was dose-dependent and more pronounced at the higher D3 dose.

In probe trial 2, testing for long term memory (LTM) one week after completion of probe trial 1, the control untreated mice spent significantly more time in the target quadrant (p = 0.00002), while the D3-treated groups did not (Fig 1B). The control untreated group spent on average 45.5% of the time in the target quadrant, while the D3 9 μg group spent less time in the target quadrant (D3 9 μg group, p = 0.016; D3 15 μg group, p = 0.21, df = 51) and significantly more time in a non-target quadrant. This indicates an LTM deficit in D3-treated mice, which is logical given that there was a STM deficit. Of note, enhanced exploratory tendencies are not an explanation to the deficits measured in the Morris Water Maze because the groups had equal performance in novel object recognition behavioral testing (S2 Fig).

These data demonstrate that in healthy young mice *acute* delivery of D3 impairs STM and LTM without causing learning deficits. This is similar to what was reported in *chronic* delivery of D3 [4]. Since *acute* or *chronic* treatments both cause memory impairment, we used D3 treatment as paradigms of drug-induced anterograde amnesia.

### Acute and chronic delivery of D3 hyperactivate TrkA in the hippocampus of healthy mice

We studied TrkA-mediated signals after acute or chronic delivery of D3, to further evaluate whether there is a correlation to TrkA actvity and memory impairment in different cholinergic brain regions.

Tissues were collected after acute delivery of D3 15 μg (n = 9) or control aCSF (n = 8) and studied for biochemical pathways relevant to memory and NGF/TrkA. Tissues were dissected from hippocampus, cortex and nucleus basalis brain regions at 1 hour (n = 1, not quantified), 5 hours (n = 4) and 24 hours (n = 4) after drug delivery and samples were studied by Western Blot using antibodies to pTrkA, pAKT, CaMKII, CREB, pErk5, pMAPK, and PKCδ. Significant changes after acute drug treatment were detected and quantified for pTrkA and CREB.

**Fig 2:**
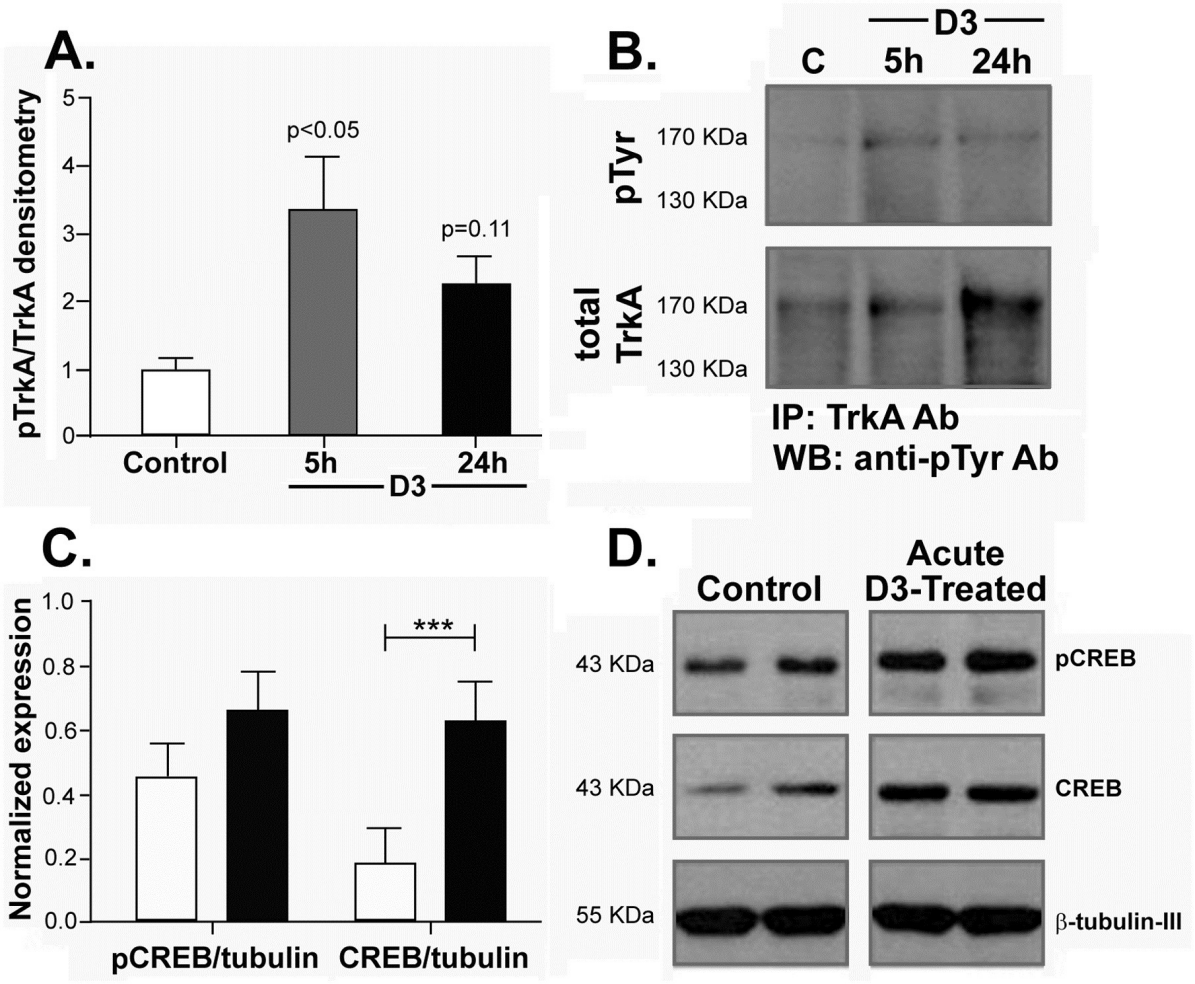
Acute D3 increases TrkA phosphorylation and CREB in the hippocampus. **(A)** Densitometric quantification of Western blots shows a significant increase in pTrkA in the hippocampus at 5 hours (p<0.05, n = 3) and a trend at 24 hours (p = 0.11, n = 2), compared to vehicle (n = 3). (**B**) Representative Western blots of samples immunoprecipitated with TrkA antibody and blotted with phospho-tyrosine antibody 4G10. Protein was extracted from the hippocampi of mice sacrificed at the times indicated. **(C)** Densitometric analysis shows a significant increase in CREB (p<0.005) at the 24-hour time point after treatment with D3 (n = 4) compared to vehicle (n = 3). **(D)** Representative Western blots of protein extracted from the hippocampi of mice sacrificed at the times indicated.

In the hippocampus of the D3-treated group there was an increase in pTrkA at 5 hours (one-between ANOVA p = 0.02, df = 5; n = 8; each with two independent technical replicate immunoprecipitations per sample). The pTrkA increase was sustained but was non-statistically significant at 24 hours (p = 0.11, df = 5; n = 8) (Fig 2A), and representative data are shown (Fig 2B). There was a significant increase in total CREB protein (p = 0.004, df = 6; n = 7) at 24 hours, without a significant increase in pCREB/CREB ratios (Fig 2C), and representative data are shown (Fig 2D). In the nucleus basalis there was increased pTrkA at 5 and 24 hours (data not shown). In the cortex, none of the proteins studied was significantly different in the acute D3 group compared to control group. Together, these data indicate that shortly after D3 treatment there is above-normal TrkA activation in the hippocampus and the nucleus basalis of young healthy mice.

**Fig 3:**
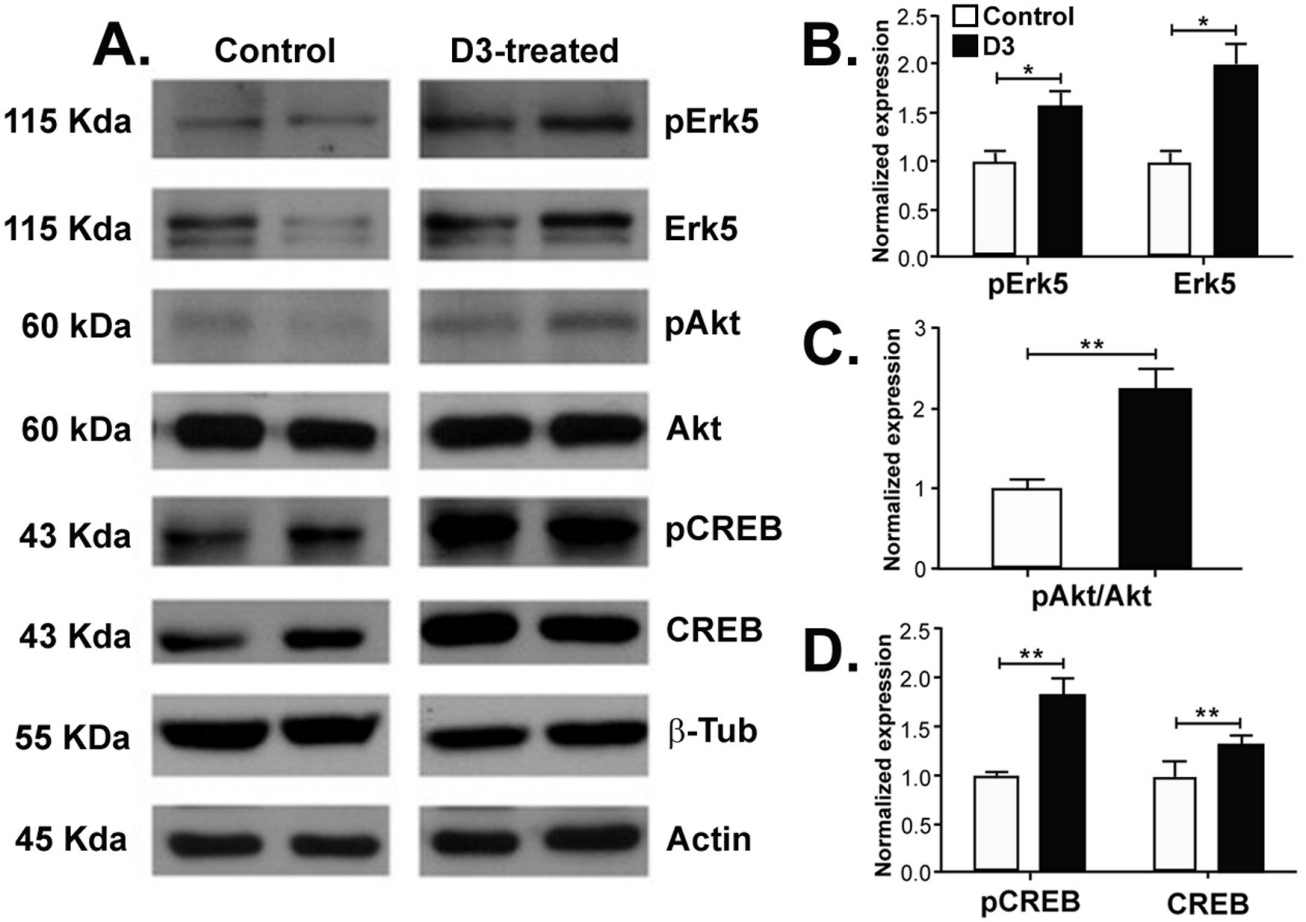
A 2-week treatment with D3 increases pErk5 Erk5, pAkt, pCREB and CREB in the hippocampus. **(A)** Representative Western Blots of proteins analyzed from hippocampus tissue. Increases in pAkt, total and pErk5, and total and pCREB were observed. Densitometry quantification of the bands shows a significant increase in **(B)** total Erk5 (p<0.05), pErk5 (p<0.05), **(C)** pAkt (p<0.01), **(D)** total CREB (p<0.01) and pCREB (p<0.01) (controls: n = 3, D3: n = 3; two repeats of Western Blots).

A comparison of control aCSF and *chronic* D3 treatment showed that five days after completion of a 2-week treatment (D3 n = 3, aCSF n = 3) there were significant changes in the hippocampus. Significant increases in pErk5 and total Erk5 (unpaired, two-tailed t-test p = 0.0089; df = 4. Fig 3A, **quantified in** Fig 3B) were detected. Total Erk5 increases could be due to relocalization from other compartments [28] or *de novo* protein translation by neurotrophic activation. Significant increases were also detected in pAkt (unpaired, two-tailed t-test p = 0.002; df = 4. Fig 3A, **quantified in** Fig 3C); and in pCREB as well as total CREB (unpaired, two-tailed t-test p = 0.0086; df = 4. Fig 3A, **quantified in** Fig 3D). In this paradigm no sustained changes were observed in any of the other proteins studied or in the other anatomical regions evaluated (cortex and nucleus basalis, data not shown).

These data indicate that signals downstream of TrkA (pErk5, Erk5, pAkt, pCREB, and CREB) are long-lived and sustained in the hippocampus for at least five days after completion of chronic D3 treatment, long after the drug is washed-off.

Next, we asked whether these signals may cause anatomical changes in hippocampus, that could lead to memory impairment in young healthy mice.

### D3 decreases dendrite branching in the basal dendrites of neurons in the CA1 region of the hippocampus in young healthy mice

We evaluated neuroanatomical changes in neuronal branching points and intersections in mice treated with D3 for two weeks compared to control.

**Fig 4:**
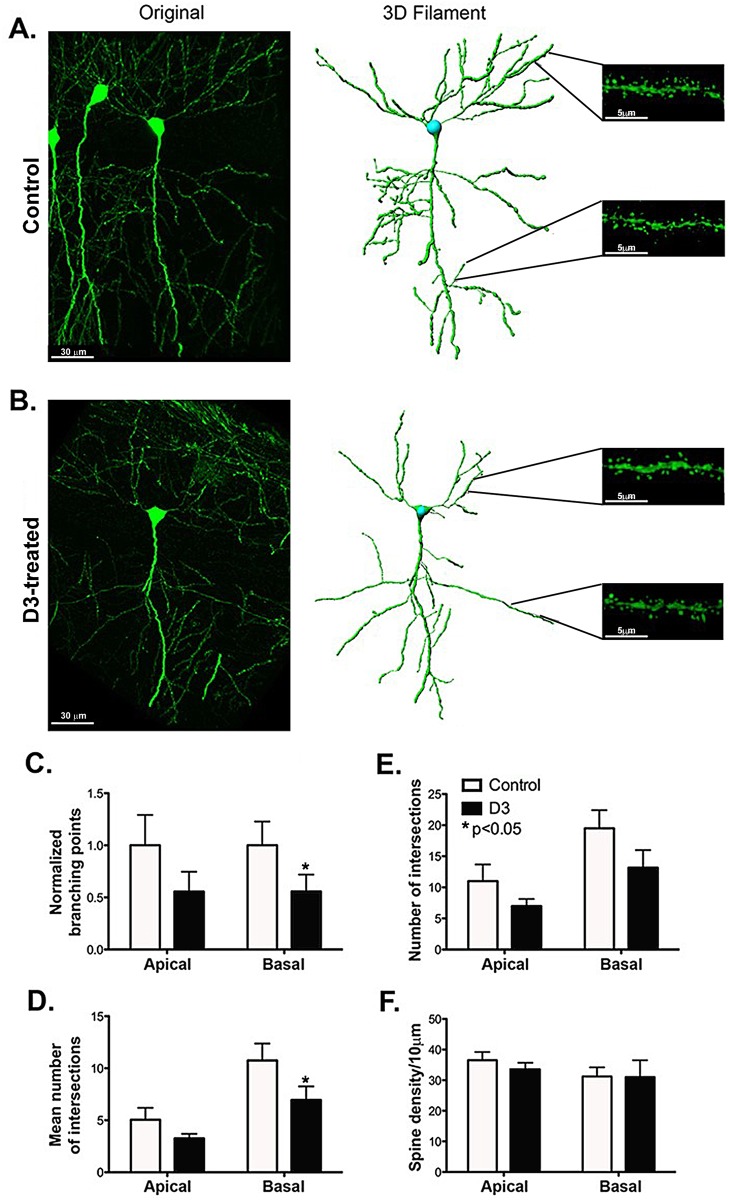
D3 reduces dendritic arborization in the basal CA1 region. **(A)** Representative confocal image, 3D filament, and magnified segments from apical and basal CA1 tertiary dendrites of an independent neuron from a control mouse. **(B)** Representative confocal image, 3D filament and magnified segments from apical and basal CA1 tertiary dendrites of an independent neuron from a 2-week D3-treated mouse. **(C)** Quantification of branching in the apical and basal region shows a decrease in branching points, significant in the basal dendrites of mice treated with D3 (p<0.05). A trend to a decrease is also observed in the apical region (p = 0.10). The values are shown as a fold decrease from control ± SEM. Total branching points of neurons belonging to D3-treated mice were normalized to the total branching points of the controls (controls n = 8, D3 n = 9, 2 cells measured per mouse, one repeat). **(D)** Mean Sholl analysis performed in the apical and basal regions yielded a significant decrease in arborization in the basal region (p<0.05). Values are shown as the average ± SEM of the mean number of intersections. **(E)** Sholl analysis performed in the apical and basal regions did not yield any statistically significant differences, but does show a trend to a decrease in arborization in the basal region (p = 0.1). Values are shown as the average ± SEM of the total number of intersections. **(F)** Spine density was quantified in the CA1 region and is expressed in spines/10μm, no significant differences were observed with D3 treatment in either of the regions.

In D3-treated mice there was a significant overall decrease in branching points in the CA1 region (ANOVA p = 0.006, df = 31; controls n = 8, D3 n = 9). The branching point decrease was more prominent in the basal branching of CA1 neurons (Newman-Keuls pairwise comparison, p = 0.021, df = 31) and a lesser and not significant decrease at the apical branching (p = 0.10, df = 31; Fig 4A versus 4B, **quantified in** Fig 4C). As well, the overall mean number of Sholl intersections per neuron were significantly decreased in both basal and apical branching in the CA1 region (ANOVA p = 0.035, df = 16). The decrease in the mean number of Sholl intersections was more prominent in the basal region (p = 0.042) than the apical region (p = 0.3, q**uantified in** Fig 4D). In addition, a trend to a lower total number of Sholl intersections was detected (ANOVA p = 0.0598, df = 16); mainly at the basal region (p = 0.10) as opposed to the apical region (p = 0.27, **quantified in** Fig 4E). In D3-treated mice, no changes in spine density (**quantified in** Fig 4F) or in mean spine length (data not shown) were detected.

**Fig 5:**
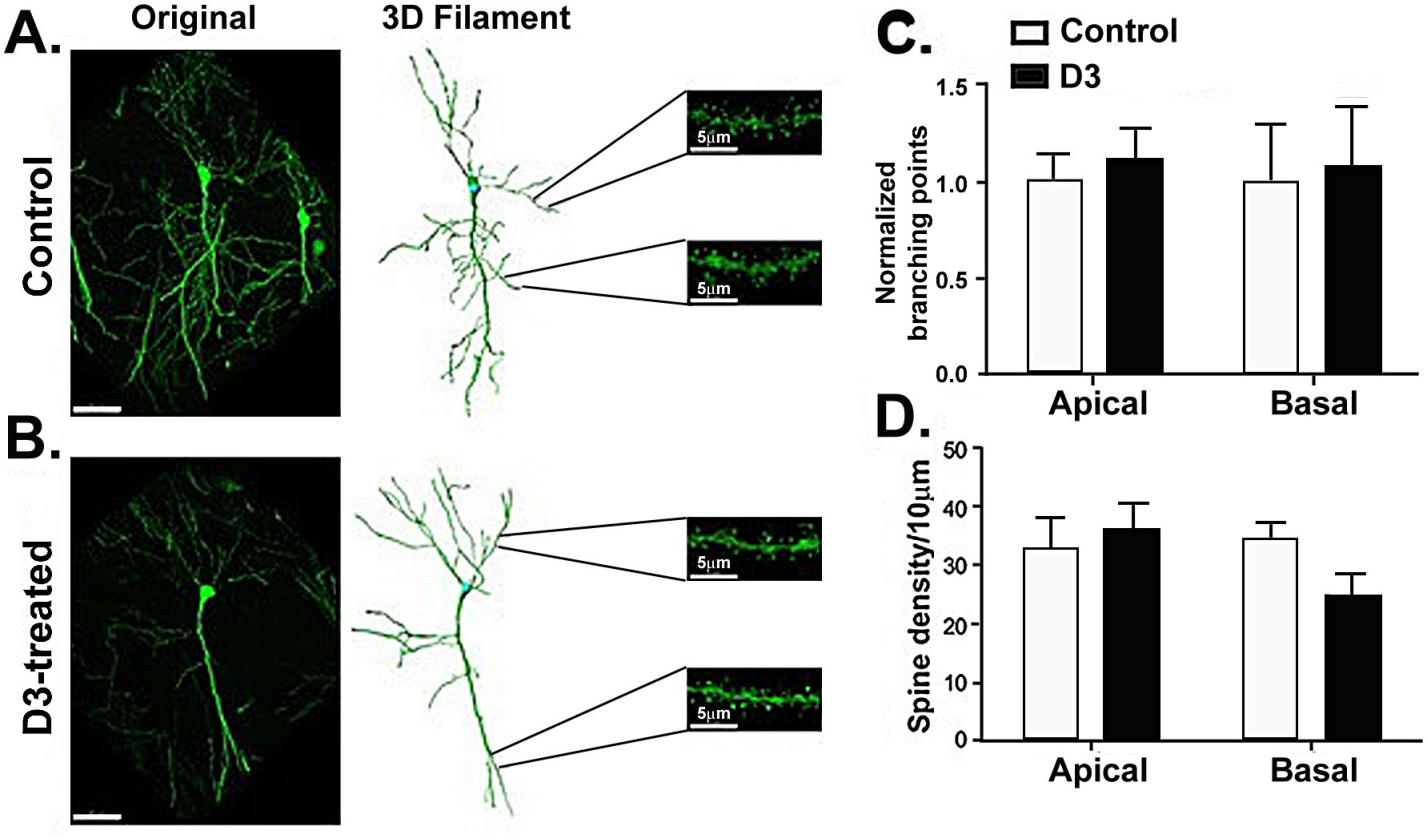
D3 has no effect on neuronal arborization in the CA3 region. **(A)** Representative confocal image, 3D filament, and magnified segments from apical and basal CA3 tertiary dendrites of an independent neuron from a control mouse. **(B)** Representative confocal image, 3D filament, and magnified segments from apical and basal CA3 tertiary dendrites of an independent neuron from a 2-week D3-treated mouse. (**C**) Quantification of branching in the apical and basal regions shows no difference between the control and the D3-treated mice (p = 0.796; controls n = 8, D3 n = 9). Total branching points of neurons belonging to D3-treated mice were normalized to the total branching points of the controls. (**D**) Spine density in the CA3 region was quantified and is expressed in spines/10μm; no significant differences were observed with D3 treatment in either of the regions.

Importantly, in the CA3 region of the hippocampus (serving as internal control) no significant changes in the amount of branching points were observed (ANOVA p = 0.796, df = 46; n = 17. Fig 5A versus 5B). There were no significant differences in branching points (**quantified in** Fig 5C) or in spine density in the CA3 region (**quantified in** Fig 5D).

Loss of branching has been associated with memory deficits, and specifically with anterograde amnesia [29]. Hence it is likely that the anterograde amnesia caused by D3-activation of TrkA-pathways is due to structural changes. The decrease in dendrite branching of the basal dendrites of neurons in the CA1 region detected after chronic D3-treatment were not due to defects in neurogenesis, as no differences were observed in BrdU+ neurons in the subgranular zone of the hippocampus (S4 Fig); though we note that neurogenesis experiments *in vivo* were not exhaustive.

In contrast to chronic D3-treatment causing structural changes, acute D3-treatment did not have an effect on hippocampal neuron branching or spine density (data not shown). This most likely is due to lack of time or lack of a sustained signal in the acute drug-treatment paradigm to allow for anatomical changes, as reported for other drugs that cause anatomical changes in hippocampus after chronic but not after acute treatment (e.g. memantine), and which may cause memory deficits [30].

Our data indicate that D3-(hyper)activation of TrkA signaling in young healthy hippocampus causes short-term and long-term signals. After acute D3 delivery, within a few hours, pTrkA and CREB are affected, and STM is impaired, but without detectable morphological changes in hippocampus. After a 2-week delivery, pErk5, pAkt, as well as increased total Erk5 and CREB are increased, and STM is impaired, along with reduced branching of the basal dendrites of CA1 neurons. This represents a previously unreported memory mechanism for TrkA action, and is consistent with the known role of CA1 neurons in memory.

### Acute D3 treatment impairs hippocampal-dependent memory consolidation

We studied the temporal and anatomical differences in acute verus chronic D3-treatment in more detail asking three questions: (a) whether memory impairment was selective for memories learned proximal to the drug-administration period, (b) whether the phenotype is due to impaired memory consolidation or to impaired memory recall, and (c) whether memory impairment is reversible once D3 is washed off while the anatomical changes persist.

**Fig 6:**
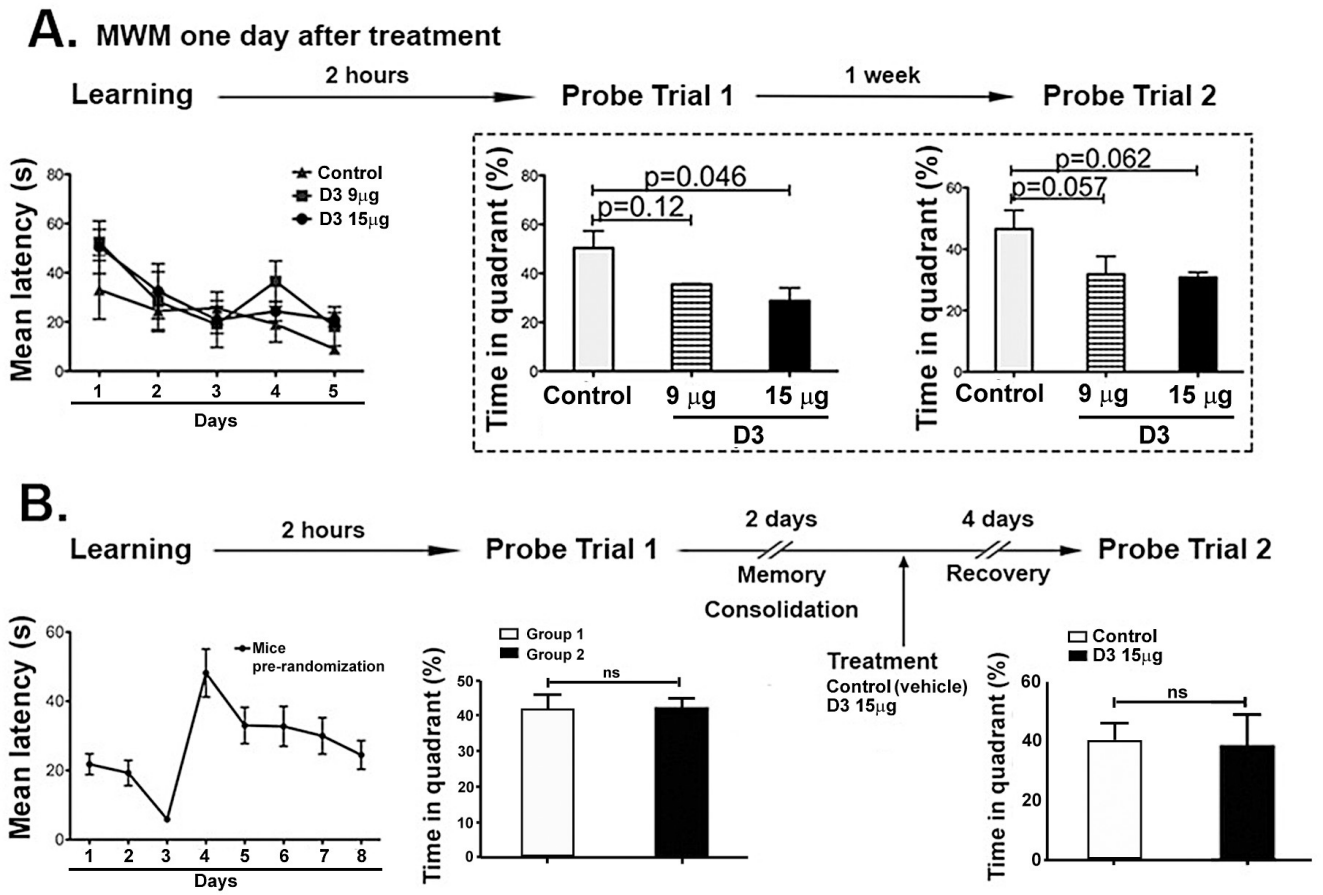
Acute D3 treatment conveys STM impairment that mainly affects hippocampal-dependent spatial memory consolidation. **(A)** Mice were trained in the Morris Water Maze (MWM) with three trials per day for 5 consecutive days. Values are shown as mean latency to find the platform (seconds). In the probe trials, values are displayed as the percentage of time the mice spent in the target quadrant. Probe Trial 1 (PT1) was performed two hours after the last trial on day 5 and Probe Trial 2 one week after PT1. Controls (n = 5), D3 9 μg (n = 3) and D3 15 μg (n = 5). Significant decreases in the percentage of time the mice spent in the target quadrant are framed in the dotted-line box. This experiment was performed twice independently, obtaining equal results. **(B)** Mice were trained as previously described and underwent PT1. They were not manipulated for the following 2 days to allow for memory consolidation. Next, they were randomly assigned to one of two groups: mice treated with vehicle (n = 6) or those treated with 15 μg of D3 (n = 5). Four days later, controls and D3-treated mice were re-tested (PT2) to assess retrieval, and no significant difference was observed, suggesting that D3 treatment impairs memory consolidation.

Memory tests starting one day after acute intraventricular delivery of D3, showed no differences in learning between the control group (n = 4), D3 9 μg group (n = 3) and D3 15 μg group (n = 5). Consistent with Fig 1, in the Probe Trial 1 (STM) and Probe Trial 2 the D3-treated groups spent a reduced percent of the time in the target quadrant as compared to the controls (Fig 6A). These data indicate that the mice treated acutely with D3 exhibited memory impairment as soon as 2 hours after the last learning trial (i.e. 5 days after drug treatment).

Given that there is no effect on learning, the impairment may be due to deficits in memory consolidation or memory retrieval. Tests were designed to evaluate these alternatives. Healthy, young mice were trained, tested to ascertain that they gained short-term spatial memory, and reposed to consolidate memory (48 hours, a period reportedly sufficient for consolidation). After randomization of the mice, one group received control aCSF (n = 6) and another group received 15 μg of D3 (n = 5), and they were re-tested for memory. No differences in memory were measured between the control and the D3-treated groups (unpaired 2-tailed t-test, p = 0.891, df = 9; control n = 6, D3 n = 5, Fig 6B).

These data suggest that once memory is consolidated, D3 does not impair memory retrieval. Hence, it is likely that D3 affects memory storage/consolidation. As spatial memory is predominantly a hippocampus-dependent process, the structural deficits observed in hippocampus CA1 neuronal branching appear to correlate with impaired spatial memory consolidation.

### The impairment in memory consolidation by acute D3-treatment is reversible

**Fig 7:**
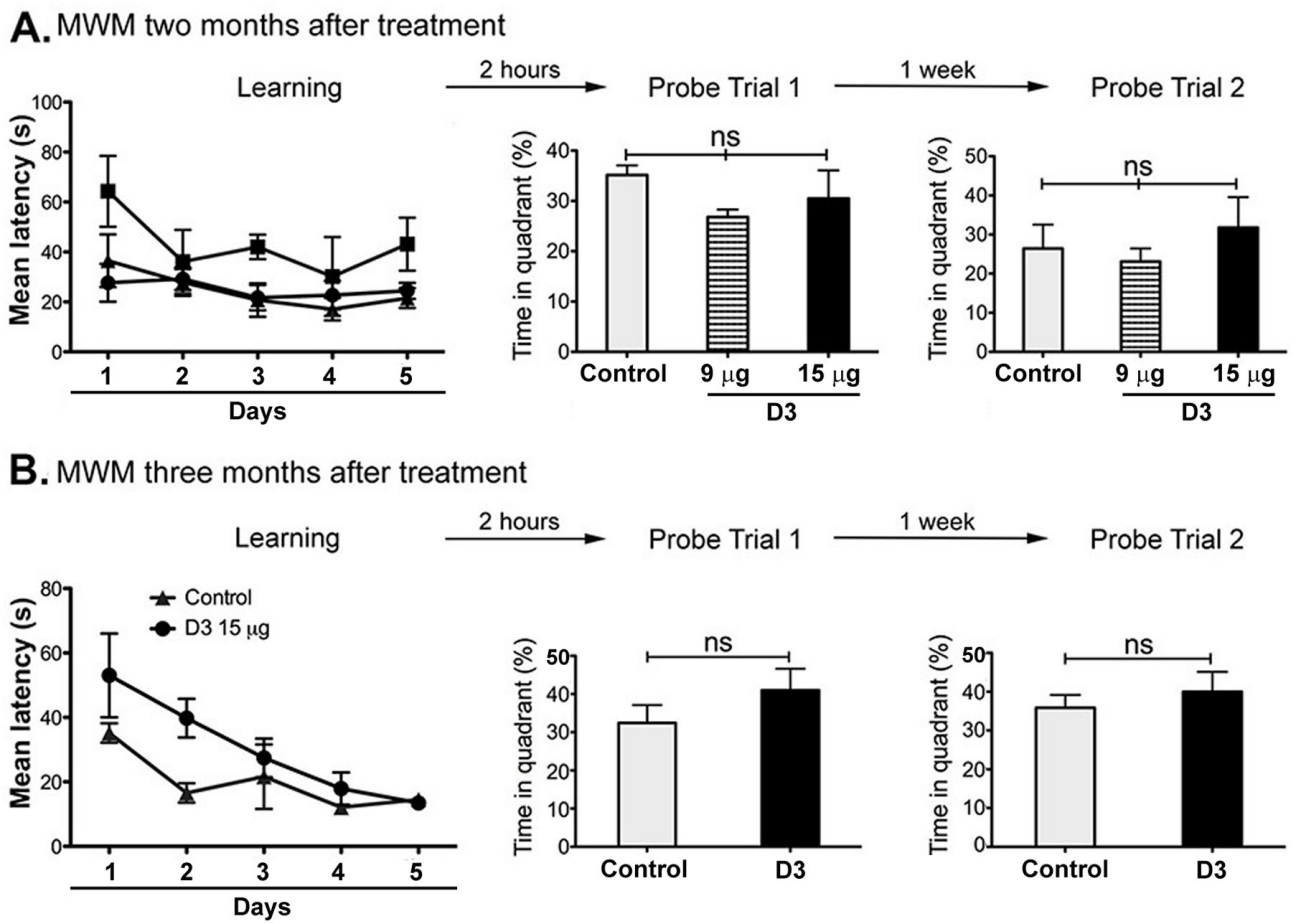
D3 impairment in STM consolidation after acute ICV injection is reversible. Mice were trained and tested through the Morris Water Maze (MWM) as previously described. Controls (n = 5), D3 9 μg (n = 3) and D3 15 μg (n = 5). No learning or memory deficits were observed **(A)** two months, nor **(B)** three monts after treatment, implying reversibility of the effects. This experiment was performed twice independently, obtaining equal results.

To test whether memory impairment was proximal to drug treatment, and/or reversible, the MWM learning and testing paradigms were repeated two and three months after acute D3 treatment. There was no memory impairment at two months post D3-treatment (p = 0.447 probe trial 1, and p = 0.682 probe trial 2, df = 10, n = 12; Fig 7A) and at three months post D3-treatment (p = 0.352 probe trial 1, and p = 0.518 probe trial 2, df = 51, n = 9; Fig 7B). All groups spent significantly more time in the target quadrant as compared to the other quadrants. These data indicate that new spatial memory tasks (learnt long after acute D3-treatment) were consolidated and recalled correctly as memory. Hence, the anterograde amnesia phenotype was reversible in the acute D3-treatment paradigm. This is in contrast with the chronic D3-treatment paradigm, which we reported was long-lasting for months after drug- wash-off [4].

## Summary

The data show that D3-intraventricular delivery leads to hyper-activation of TrkA signals both in the acute and the chronic treatment paradigms, and a behavioral phenotype reminiscent of anterograde amnesia. Acute or chronic D3-treatment of young healthy mice conveys memory impairment due to failure in memory consolidation, in a time period proximal to drug action, which in the acute paradigm is reversible but in the chronic paradigm is sustained long after drug-wash-off. In the acute treatment paradigm the memory deficits are reversible, and there are no detectable anatomical changes in hippocampus. In the chronic treatment paradigm the memory deficits are sustained, this is correlated with and likely due to a decrease in hippocampus CA1 neuronal branching. Table 1 summarizes a comparison of the data obtained after acute and chronic delivery of the pharmacological TrkA agonist D3.

**Table 1:** Summary of data.

ICV Treatment	Biochemical changes	Structural changes	Electrophys.	Behavior
Acute	pTrkA and CREB increase in the hippocampus	None	No changes in LTP	Reversible STM and LTM impairment in MWM, without affecting learning. No effect in NOR
Chronic * (2 weeks)	pErk, Erk5, pCREB, CREB, pAKT* and pro-BDNF* increase in the hippocampus Increase NRH-2 in cortex and hippocampus*	Decreased branching in the basal dendrites of neurons in the CA1 region of the hippocampus	Increased LTD	Long-lasting, LTM impairment in MWM, without affecting learning or STM

* Some results previously published by Aboulkassim et al.

STM: short-term memory; LTM: long-term memory; MWM: Morris Water Maze; NOR: Novel Object Recognition; LTP: long-term potentiation. LTD: long-term depression.

## Discussion

Reportedly, loss of TrkA density/activity correlates with cholinergic neuronal atrophy and death, and with disease progression in in rodent models of memory impairment [12] and humans [14, 15, 31]. These observations led to the hypothesis that selective activation of TrkA may be neuroprotective and beneficial to memory [17], a concept that has been proven experimentally in animal models of disease [4, 22].

However, paradoxically, hyperactivation of TrkA in healthy mice led to memory impairment. Here, we show that in healthy young mice, the chronic (2-week) intraventricular delivery of the TrkA agonist D3 caused hyperactivation of TrkA-mediated signals in the hippocampus, with a decrease in CA1 neuronal arborization, and an *irreversible* spatial memory deficit. In healthy young mice, an acute delivery of the TrkA agonist D3 caused hyperactivation of TrkA in the BFCN and the hippocampus, without any detectable structural changes in CA1, and a *reversible* spatial memory deficit. Memory deficits are linked to impaired memory consolidation and are not due to impaired learning or memory retrieval. These data are an example of a drug that is beneficial in disease states may be detrimental in healthy states. Table 1 summarizes data obtained upon intraventricular acute or chronic delivery of the pharmacological TrkA agonist D3.

### TrkA-dependent signaling pathways, relevant to memory, are activated by D3 in hippocampus

TrkA is mainly expressed in the hippocampus and the BCFN, and expectedly biochemical changes would take place in these anatomical regions. Indeed, after acute D3-treatment, TrkA-biochemical signals were detected in the hippocampus and nucleus basalis. Nonetheless, 5 days after completion of a chronic treatment (a 2-week course), sustained increases in TrkA signaling pathways were only observed in the hippocampus. No significant alterations in biochemical signals were detected by quantitative western blot techniques in the cortex, though this could be due to dilution of the signal.

The increases observed in Erk5 at different time-points and in different regions of the brain, follow described models of D3 tissue penetration [22] and TrkA activation either directly in the cell body or in neuronal terminals [32, 33]. Such increase in activity in Erk pathways could lead to altered dendritogenesis and synaptogenesis [34].

The increase in Akt phosphorylation, especially in the hippocampus, was previously described by our group [4], such an increase can lead to hippocampal long-term depression (LTD) through deregulation of the PI3K/Akt pathway. CREB and its active phosphorylated form are increased in a sustained fashion, particularly in the hippocampus. CREB acts as a transcription factor for many proteins that mediate survival and differentiation. D3-mediated increases in protein expression could be due to the increased transcription or stability, which explains physiological effects outlasting drug washout.

### D3-mediated pharmacological effects

NGF is produced in the cortex, basal forebrain and hippocampus (8). Hippocampal homeostasis depends on connections from basal cholinergic neurons (BFCN)—which innervate the cortex and hippocampus (5). BFCN depend on the proNGF/NGF ratio and the p75/TrkA balance [7, 35]. Deficiency of TrkA expression/function, or deficiency of NGF transport, or maturation of NGF from proNGF have been linked to BFCN degeneration [36], as well as memory deficits both in animal models and dementia patients [14]. On the other hand, a conditional knockout of the NGF/TrkA axis in the young adult was not sufficient to cause learning/memory impairment [37]. This means that the impact of the NGF/TrkA axis in memory depends on age and health state. Once activated, the signals downstream of TrkA may convey memory enhancement or memory impairment depending on the health state and baseline TrkA activation state of the cholinergic neurons, and the levels of TrkA expression.

In the present work, the drug kinetics, and drug target selectivity are also important. D3 is a selective TrkA partial agonist, that can potentiate the biological effects of low levels of NGF, and does not bind to p75 [4, 21, 22, 38]. However, unbound p75 receptors can have a negative impact on TrkA activation, and when p75 is bound it can have either a positive or a negative impact depending on which ligand binds to it [39]. Hence, it is important to consider that in healthy and in diseased brains memory can be impacted by different ligand-receptor interactions such the proNGF/p75 axis, and the presence of other p75 ligands, co-receptors, co-factors, or low levels of mature NGF.

### The hippocampus as a likely the target for D3-mediated memory impairment

Cholinergic basal forebrain neurons are connected through the entorhinal cortex to the basal region of the CA1 area [40], hence the decrease in dendrite branching in pyramidal neurons in the CA1 region may be Place Cells [41, 42], and this may account for the spatial memory defects.

Chronic D3 administration caused LTM impairment, without causing STM or learning deficits. Acute D3 administration conveyed not only LTM but also STM impairment without causing learning deficits. This is curious given that both STM and LTM require the hippocampus for initial processing [43], and STM takes place in a short span from minutes to hours [44]. This exemplifies how different drug regimens (a continuous low dose versus an acute higher dose) may cause different physiological effects, but also informs as to the differences in TrkA-mechanisms that control learning and remembering in the short term versus the long term.

Drug-induced learning impairment is not observed during the 5-day learning phase which has inter-trial spans of 45 minutes, likely due to synaptic tagging and strengthened synapses during each learning trial [45]. Drug-induced memory impairment is evident in probe 1 (two hours after the last learning trial). During the probe trials, the platform is no longer present, the mouse does not find the platform and one may hypothesize the synapse is not strengthened in the D3-treated mice. Instead, there may be a faster weakening or disruption of the synapses, and therefore weakening of memory consolidation. We did not observe structural changes in the hippocampus of mice that underwent acute D3 injections; however there may still be a transient effect on synaptic stability. The transient nature of these changes would explain their reversibility. Reversibility also indicates that the negative impact on memory consolidation in healthy mice is not due simply to off-target toxicity or to non-specific effects; and this view is further supported by the *beneficial* effect of chronic D3-treatment in rodent models of AD and ageing. Conversely, in mice that had chronic treatment with D3, we observed a decrease in CA1 neuronal arborization, which can lead to increased long term depression (LTD) [4], without changes in LTP (which we tested both in chronic and acute D3 treatments, S3 Fig).

These structural changes may underlie the irreversibility of the memory impairment observed with chronic administration of D3. In contrast, the deficits observed with acute D3 administration are reversible, and no structural changes are detected. Reversibility indicates that the negative impact on memory consolidation in healthy mice is not due simply to off-target toxicity or to non-specific effects; and this view is further supported by the *beneficial* effect of chronic D3-treatment in rodent models of AD and ageing.

Previous reports have shown both reversible and irreversible processes associated with memory impairment. Pharmacological agents such as opioids cause reversible anterograde amnesia [46]. Anatomical disruptions cause irreversible anterograde amnesia [47]; and even Alzheimer’s dementia could be viewed as a form of irreversible anterograde amnesia. Anterograde amnesia is the inability to transfer information from the short-term store into the long-term store, with the hippocampus CA1 region being implicated. With D3 the ability to learn is retained, but the ability to form new memories is negatively affected through an impairment in hippocampal-mediated consolidation. The differences observed in consolidation versus retrieval may be due to the hippocampus seemingly having a greater role in consolidation of memories as opposed to retrieval [48].

It was somewhat surprising to find no significant differences in spine density, mean spine volume and mean spine length. This could be explained by the formation of asymmetric shaft synapses, with excitatory synapses retracting back within the dendrite [49]. Nonetheless, even without a decrease in spine density per unit length, a decrease in dendritic arborization would cause an overall decrease in the total number of spines.

Hippocampal neurogenesis also reportedly affects memory phenotypes [50], but at the doses tested D3 had no significant effects on hippocampal neurogenesis *in vivo* (S4 Fig). Hence, TrkA/NGF do not appear to mediate neurogenesis *in vivo*. However, *ex vivo* D3 promoted growth and survival of cell lines [21] and promoted neurogenesis of embryonic hippocampal primary cultures (S5 Fig), suggesting that *in vivo* in the adult there may be mechanisms that modulate D3-promoted or TrkA-mediated neurogenesis of subgranular zone neurons.

In summary, D3 is a TrkA partial agonist which when administered acutely to healthy young mice causes a reversible impairment in hippocampal-dependent memory consolidation. The irreversible long-term memory impairment observed after a 2-week delivery is likely due to a decrease in pyramidal neuron differentiation in the CA1 region of the hippocampus. This work expands on the role of TrkA in memory networks, and the different consequences of TrkA activation in healthy versus disease states, and may contribute to the rationalization of new therapeutic strategies.

## Supporting information

S1 Fig Timeline of the chronic and acute drug-treatments and experimental endpoints. (TIF)

S2 Fig D3 does not have an effect on habituation or object exploration. In all graphs, the first three days correspond to habituation, the following two days to familiarization (identical objects) and finally on the fifth day the novel object trial. A) The distance travelled by the mice was recorded in meters (p = 0.7). B) The time spent moving was recorded in seconds (out of a total of 300 s, p = 0.4). C) The percentage of the field explored by the mice (p = 0.9). D) The number of entries to the different quadrants in the field (p = 0.7). No significant differences were observed in any of the measurements (Controls n = 6, D3 n = 7; one repeat). Before the novel object recognition (NOR) test, mice were allowed to habituate to the testing environment. There were no differences between the controls and the D3-treated mice in the distance (path) traveled, the time spent exploring, the percentage of the space the mouse covered or the amount of times the mouse entered the different sections of the field. The same measurements were performed during object exploration and again we observed no differences between the two experimental groups. Both groups increased exploration when exposed to the objects for the first time and when exposed to the novel object (Controls n = 6, D3 n = 7).
(TIF)

S3 Fig Acute D3 treatment has no effects on LTP. (A) Electrophysiological recordings were performed in mice 6 days after acute ICV injection with D3 or vehicle. (B) No significant differences were observed in percentage potentiation during the last 10 minutes, at 60 minutes after tetanus, a parameter corresponding to LTP (Controls n = 7 recordings, D3 n = 6 recordings; one repeat). To test whether D3 conveyed changes in LTP and baseline connectivity after acute ICV injection, electrophysiological analysis was performed. No differences were observed in LTP between D3 and vehicle-treated wild type mice, 6 days after acute ICV injections. No differences were observed in the input/output analysis either (data not shown).
(TIF)

S4 Fig Chronic D3 delivery *in vivo* has no effect in vivo on neurogenesis in the SGZ of the dentate gyrus. Mice were treated with aCSF or D3 (40 μg) ICV, simultaneously with BrdU PO. Immunofluorescence was performed for BrdU and NeuN. The amount of BrdU positive neurons was quantified in the subgranular zone of the dentate gyrus. No significant differences were observed between the controls (n = 2) and the D3-treated (n = 2) mice (p = 0.16). Conceivably, the decreases in dendrite branching in the CA1 region detected after D3-treatment may be caused by or coincide with defects in neurogenesis. Mice received chronic D3 ICV for 2 weeks, as well as, during the same period, BrdU in the water to label dividing cells. A non-statistically significant decrease in BrdU positive neurons was observed in the D3-treated mice (201±34 BrdU-positive neurons, n = 2) compared to controls (295±27 BrdU-positive neurons, n = 2) in the subgranular zone of the dentate gyrus of the hippocampus (unpaired 2-tailed t-test, p = 0.16, df = 2; n = 4 mice, approximately 30 sections per mouse, one repeat). The decrease in dendrite branching of the basal dendrites of neurons in the CA1 region was therefore independent of detectable effects on neurogenesis. BrdU Labeling in vivo, and Analysis. To study neurogenesis in vivo, BrdU was delivered at a concentration of 1 mg/mL in 1% glucose in drinking water. Mice were housed individually with individual water bottles containing BrdU, and the water consumed was constant between groups. BrdU was administered for the 2 weeks that the mice were administered chronically with D3 or aCSF. The mice were then perfused and their brains were fixed (4% PFA), processed for OCT, and cryo-sectioned into 12 μm thick slices (LEICA (Concord, Canada) 3050s cryostat). BrdU was exposed by submerging slides in 1N HCl at 45°C for 30 minutes. Cell membranes were permeabilized with 0.4% Triton X-100 PBS. Tissues were blocked with 5% NGS/3% BSA for 1 hour at room temperature. Slides were incubated with BrdU antibody 1:300 (Abcam ab6326) and NeuN 1:500 (Millipore mab-N78) in 0.2% Triton X-100 PBS overnight at 4°C. After washing (0.2% Triton X-100 PBS), secondary antibodies (goat anti-rat FITC 1:1,000 and goat anti-mouse Alexa 594 1:1,000) were incubated in 0.2% Triton X-100 PBS at room temperature for 45 minutes. Sections were washed and covered with VECTASHIELD (Vectorlabs) mounting medium, and visualized under an epifluorescence microscope. Approximately 30 sections per brain spanning the entire hippocampus were quantified manually using Image J cell counter.
(TIF)

S5 Fig D3 significantly increases hippocampal neurogenesis *in vitro*. A) Primary embryonic hippocampal cultures were treated with supplemented Neurobasal medium (Control), and the following compounds in the same medium: C59 (an inert compound similar to D3), D3 at two different concentrations: 10 μM and 1 μM, and NGF at two different concentrations: 1 nM and 100 pM. Immunofluorescence was performed for Ki-67 (red) and MAP2 (green). B) Quantification of the proportion of Ki-67 positive neurons showed a significant increase in neurogenesis with D3 at both concentrations (*p* = 0.06 and *p*<0.05) and with NGF at 1 nM (*p*<0.05), two independent repeats.
(TIF)

## Abbreviations

aCSF: artificial cerebrospinal fluid
AD: Alzheimer’s disease
Akt: protein kinase B
APP: amyloid precursor protein
BFCN: basal forebrain cholinergic neuron
BrdU: bromodeoxyuridine
CaMKII: Ca2+/calmodulin-dependent protein kinase II
CREB: cAMP response element-binding protein
Erk5: extracellular signal-regulated kinase 5
GFP: green fluorescent protein
ICV: intracerebroventricular
LTD: long-term depression
LTM: long-term memory
LTP: long-term potentiation
MAPK: mitogen-activated protein kinase
MCI: mild cognitive impairment
MWM: Morris water maze
NGF: nerve growth factor
PKCδ: protein kinase C zeta
PT1-2: probe trial 1–2
SGZ: subgranular zone
STM: short-term memory
TrkA: tropomyosin receptor kinase A

## References

[1] Mendiola-Precoma J, Berumen L, Padilla K, Garcia-Alcocer G. Therapies for prevention and treatment of Alzheimer’s disease. BioMed Research International. 2016;2016. 10.1155/2016/2589276 PMC4980501 27547756

[2] Duan L, Bhattacharyya BJ, Belmadani A, Pan L, Miller RJ, Kessler JA. Stem cell derived basal forebrain cholinergic neurons from Alzheimer’s disease patients are more susceptible to cell death. Molecular neurodegeneration. 2014;9:3 10.1186/1750-1326-9-3 . 24401693 PMC3896712

[3] Kandel ER, Dudai Y, Mayford MR. The molecular and systems biology of memory. Cell. 2014;157(1):163–86. 10.1016/j.cell.2014.03.001 . 24679534

[4] Aboulkassim T, Tong XK, Tse YC, Wong TP, Woo SB, Neet KE, et al Ligand-dependent TrkA activity in brain differentially affects spatial learning and long-term memory. Molecular pharmacology. 2011;80(3):498–508. 10.1124/mol.111.071332 . 21616921

[5] Niewiadomska G, Mietelska-Porowska A, Mazurkiewicz M. The cholinergic system, nerve growth factor and the cytoskeleton. Behavioural brain research. 2011;221(2):515–26. 10.1016/j.bbr.2010.02.024 . 20170684

[6] Deinhardt K, Chao MV. Trk receptors. Handbook of experimental pharmacology. 2014;220:103–19. 10.1007/978-3-642-45106-5_5 . 24668471

[7] Magno L, Kretz O, Bert B, Ersözlü S, Vogt J, Fink H, et al The integrity of cholinergic basal forebrain neurons depends on expression of Nkx2‐1. European Journal of Neuroscience. 2011;34(11):1767–82. 10.1111/j.1460-9568.2011.07890.x 22098391 PMC3486784

[8] Bruno MA, Cuello AC. Activity-dependent release of precursor nerve growth factor, conversion to mature nerve growth factor, and its degradation by a protease cascade. Proc Natl Acad Sci U S A. 2006;103(17):6735–40. 10.1073/pnas.0510645103 . 16618925 PMC1458950

[9] Iulita MF, Cuello AC. Nerve growth factor metabolic dysfunction in Alzheimer’s disease and Down syndrome. Trends in pharmacological sciences. 2014;35(7):338–48. 10.1016/j.tips.2014.04.010 24962069

[10] Scott SA, Mufson EJ, Weingartner JA, Skau KA, Crutcher KA. Nerve growth factor in Alzheimer’s disease: increased levels throughout the brain coupled with declines in nucleus basalis. The Journal of neuroscience: the official journal of the Society for Neuroscience. 1995;15(9):6213–21. . 7666203 10.1523/JNEUROSCI.15-09-06213.1995 PMC6577665

[11] Salehi A, Delcroix J-D, Belichenko PV, Zhan K, Wu C, Valletta JS, et al Increased App expression in a mouse model of Down’s syndrome disrupts NGF transport and causes cholinergic neuron degeneration. Neuron. 2006;51(1):29–42. 10.1016/j.neuron.2006.05.022 16815330

[12] Saragovi HU. Progression of age-associated cognitive impairment correlates with quantitative and qualitative loss of TrkA receptor protein in nucleus basalis and cortex. Journal of neurochemistry. 2005;95(5):1472–80. 10.1111/j.1471-4159.2005.03479.x . 16219032

[13] Debeir T, Saragovi HU, Cuello AC. A nerve growth factor mimetic TrkA antagonist causes withdrawal of cortical cholinergic boutons in the adult rat. Proc Natl Acad Sci U S A. 1999;96(7):4067–72. Epub 1999/03/31. 10.1073/pnas.96.7.4067 . 10097164 PMC22421

[14] Mufson EJ, Ma SY, Cochran EJ, Bennett DA, Beckett LA, Jaffar S, et al Loss of nucleus basalis neurons containing trkA immunoreactivity in individuals with mild cognitive impairment and early Alzheimer’s disease. The Journal of comparative neurology. 2000;427(1):19–30. . 11042589 10.1002/1096-9861(20001106)427:1<19::aid-cne2>3.0.co;2-a

[15] Counts SE, Nadeem M, Wuu J, Ginsberg SD, Saragovi HU, Mufson EJ. Reduction of cortical TrkA but not p75(NTR) protein in early-stage Alzheimer’s disease. Annals of neurology. 2004;56(4):520–31. 10.1002/ana.20233 . 15455399

[16] Ferreira D, Westman E, Eyjolfsdottir H, Almqvist P, Lind G, Linderoth B, et al Brain changes in Alzheimer’s disease patients with implanted encapsulated cells releasing nerve growth factor. Journal of Alzheimer’s Disease. 2015;43(3):1059–72. 10.3233/JAD-141068 25147108

[17] Josephy-Hernandez S, Jmaeff S, Pirvulescu I, Aboulkassim T, Saragovi HU. Neurotrophin receptor agonists and antagonists as therapeutic agents: An evolving paradigm. Neurobiology of Disease. 2017;97:139–55. 10.1016/j.nbd.2016.08.004 27546056

[18] Bai Y, Dergham P, Nedev H, Xu J, Galan A, Rivera JC, et al Chronic and acute models of retinal neurodegeneration TrkA activity are neuroprotective whereas p75NTR activity is neurotoxic through a paracrine mechanism. Journal of Biological Chemistry. 2010;285(50):39392–400. 10.1074/jbc.M110.147801 20943663 PMC2998128

[19] Saragovi HU, Zaccaro MC. Small molecule peptidomimetic ligands of neurotrophin receptors, identifying binding sites, activation sites and regulatory sites. Current pharmaceutical design. 2002;8(24):2201–16. . 12369863 10.2174/1381612023393215

[20] Longo FM, Massa SM. Small-molecule modulation of neurotrophin receptors: a strategy for the treatment of neurological disease. Nature reviews Drug discovery. 2013;12(7):507–25. . 23977697 10.1038/nrd4024

[21] Maliartchouk S, Feng Y, Ivanisevic L, Debeir T, Cuello AC, Burgess K, et al A designed peptidomimetic agonistic ligand of TrkA nerve growth factor receptors. Molecular pharmacology. 2000;57(2):385–91. . 10648649

[22] Bruno MA, Clarke PB, Seltzer A, Quirion R, Burgess K, Cuello AC, et al Long-lasting rescue of age-associated deficits in cognition and the CNS cholinergic phenotype by a partial agonist peptidomimetic ligand of TrkA. The Journal of neuroscience: the official journal of the Society for Neuroscience. 2004;24(37):8009–18. 10.1523/JNEUROSCI.1508-04.2004 . 15371501 PMC6729798

[23] DeVos SL, Miller TM. Direct intraventricular delivery of drugs to the rodent central nervous system. Journal of visualized experiments: JoVE. 2013;(75):e50326 10.3791/50326 . 23712122 PMC3679837

[24] Bonifacino JS, Dell’Angelica EC, Springer TA. Immunoprecipitation. Current protocols in molecular biology / edited by Frederick M Ausubel [et al]. 2001;Chapter 10:Unit 10 6. 10.1002/0471142727.mb1016s48 . 18265056

[25] Feng G, Mellor RH, Bernstein M, Keller-Peck C, Nguyen QT, Wallace M, et al Imaging neuronal subsets in transgenic mice expressing multiple spectral variants of GFP. Neuron. 2000;28(1):41–51. . 11086982 10.1016/s0896-6273(00)00084-2

[26] Swanger SA, Yao X, Gross C, Bassell GJ. Automated 4D analysis of dendritic spine morphology: applications to stimulus-induced spine remodeling and pharmacological rescue in a disease model. Molecular brain. 2011;4:38 10.1186/1756-6606-4-38 . 21982080 PMC3213078

[27] Morris R. Developments of a water-maze procedure for studying spatial learning in the rat. Journal of neuroscience methods. 1984;11(1):47–60. . 6471907 10.1016/0165-0270(84)90007-4

[28] van Oterendorp C, Sgouris S, Schallner N, Biermann J, Lagreze WA. Retrograde neurotrophic signaling in rat retinal ganglion cells is transmitted via the ERK5 but not the ERK1/2 pathway. Invest Ophthalmol Vis Sci. 2014;55(2):658–65. Epub 2014/01/09. 10.1167/iovs.13-12985 . 24398098

[29] Zola-Morgan S, Squire LR, Amaral D. Human amnesia and the medial temporal region: enduring memory impairment following a bilateral lesion limited to field CA1 of the hippocampus. Journal of Neuroscience. 1986;6(10):2950–67. 3760943 10.1523/JNEUROSCI.06-10-02950.1986 PMC6568782

[30] Sekar S, Jonckers E, Verhoye M, Willems R, Veraart J, Van Audekerke J, et al Subchronic memantine induced concurrent functional disconnectivity and altered ultra-structural tissue integrity in the rodent brain: revealed by multimodal MRI. Psychopharmacology (Berl). 2013;227(3):479–91. 10.1007/s00213-013-2966-3 . 23354531

[31] Sendera T, Ma S, Jaffar S, Kozlowsk P, Kordower J, Mawal Y, et al Reduction in TrkA-immunoreactive neurons is not associated with an overexpression of galaninergic fibers within the nucleus basalis in Down’s syndrome. J Neurochem. 2000;74(3):1185–96. 10693951 10.1046/j.1471-4159.2000.741185.x

[32] Watson FL, Heerssen HM, Bhattacharyya A, Klesse L, Lin MZ, Segal RA. Neurotrophins use the Erk5 pathway to mediate a retrograde survival response. Nature neuroscience. 2001;4(10):981–8. 10.1038/nn720 11544482

[33] Pan YW, Storm DR, Xia Z. Role of adult neurogenesis in hippocampus-dependent memory, contextual fear extinction and remote contextual memory: new insights from ERK5 MAP kinase. Neurobiology of learning and memory. 2013;105:81–92. 10.1016/j.nlm.2013.07.011 . 23871742 PMC3782100

[34] Wang W, Pan YW, Zou J, Li T, Abel GM, Palmiter RD, et al Genetic activation of ERK5 MAP kinase enhances adult neurogenesis and extends hippocampus-dependent long-term memory. The Journal of neuroscience: the official journal of the Society for Neuroscience. 2014;34(6):2130–47. 10.1523/JNEUROSCI.3324-13.2014 . 24501354 PMC3913867

[35] Fortress AM, Buhusi M, Helke KL, Granholm A-CE. Cholinergic degeneration and alterations in the TrkA and p75NTR balance as a result of Pro-NGF injection into aged rats. Journal of aging research. 2011;2011. 10.4061/2011/460543 PMC3140182 21785728

[36] Sanchez-Ortiz E, Yui D, Song D, Li Y, Rubenstein JL, Reichardt LF, et al TrkA gene ablation in basal forebrain results in dysfunction of the cholinergic circuitry. Journal of Neuroscience. 2012;32(12):4065–79. 10.1523/JNEUROSCI.6314-11.2012 22442072 PMC3403817

[37] Müller M, Triaca V, Besusso D, Costanzi M, Horn JM, Koudelka J, et al Loss of NGF-TrkA signaling from the CNS is not sufficient to induce cognitive impairments in young adult or intermediate-aged mice. Journal of Neuroscience. 2012;32(43):14885–98. 10.1523/JNEUROSCI.2849-12.2012 23100411 PMC6704821

[38] Zaccaro MC, Lee HB, Pattarawarapan M, Xia Z, Caron A, L’Heureux PJ, et al Selective small molecule peptidomimetic ligands of TrkC and TrkA receptors afford discrete or complete neurotrophic activities. Chemistry & biology. 2005;12(9):1015–28. 10.1016/j.chembiol.2005.06.015 . 16183026

[39] Maliartchouk S, Saragovi HU. Optimal nerve growth factor trophic signals mediated by synergy of TrkA and p75 receptor-specific ligands. The Journal of neuroscience: the official journal of the Society for Neuroscience. 1997;17(16):6031–7. . 9236214 10.1523/JNEUROSCI.17-16-06031.1997 PMC6568372

[40] Mufson EJ, Counts SE, Perez SE, Ginsberg SD. Cholinergic system during the progression of Alzheimer’s disease: therapeutic implications. Expert review of neurotherapeutics. 2008;8(11):1703–18. 10.1586/14737175.8.11.1703 . 18986241 PMC2631573

[41] O’Keefe J, Conway DH. Hippocampal place units in the freely moving rat: why they fire where they fire. Experimental brain research. 1978;31(4):573–90. . 658182 10.1007/BF00239813

[42] Moser MB, Rowland DC, Moser EI. Place Cells, Grid Cells, and Memory. Cold Spring Harbor perspectives in biology. 2015;7(2). 10.1101/cshperspect.a021808 . 25646382 PMC4315928

[43] Milner B, Corkin S, Teuber H-L. Further analysis of the hippocampal amnesic syndrome: 14-year follow-up study of HM. Neuropsychologia. 1968;6(3):215–34.

[44] Buchsbaum B, D’Esposito M. Short-term and working memory systems. Learning and memory: A comprehensive reference. 2008:237–2690.

[45] Martin KC, Kosik KS. Synaptic tagging—who’s it? Nature reviews Neuroscience. 2002;3(10):813–20. 10.1038/nrn942 . 12360325

[46] Sharifi KA, Rezayof A, Torkaman-Boutorabi A, Zarrindast M-R. The major neurotransmitter systems in the basolateral amygdala and the ventral tegmental area mediate morphine-induced memory consolidation impairment. Neuroscience. 2017;353:7–16. 10.1016/j.neuroscience.2017.03.036 28412500

[47] Nadel L, Moscovitch M. Memory consolidation, retrograde amnesia and the hippocampal complex. Curr Opin Neurobiol. 1997;7(2):217–27. . 9142752 10.1016/s0959-4388(97)80010-4

[48] Abel T, Lattal KM. Molecular mechanisms of memory acquisition, consolidation and retrieval. Curr Opin Neurobiol. 2001;11(2):180–7. . 11301237 10.1016/s0959-4388(00)00194-x

[49] Bourne JN, Harris KM. Coordination of size and number of excitatory and inhibitory synapses results in a balanced structural plasticity along mature hippocampal CA1 dendrites during LTP. Hippocampus. 2011;21(4):354–73. 10.1002/hipo.20768 . 20101601 PMC2891364

[50] Birch AM, Kelly ÁM. Chronic intracerebroventricular infusion of nerve growth factor improves recognition memory in the rat. Neuropharmacology. 2013;75:255–61. 10.1016/j.neuropharm.2013.07.023 23932816

