# Supplementary figures and images for: Pharmacological interrogation of TrkA-mediated mechanisms in hippocampal-dependent memory consolidation
Source: PLoS One. 2019 Jun 24;14(6):e0218036. doi: 10.1371/journal.pone.0218036 (PMC6590805; doi:10.1371/journal.pone.0218036)

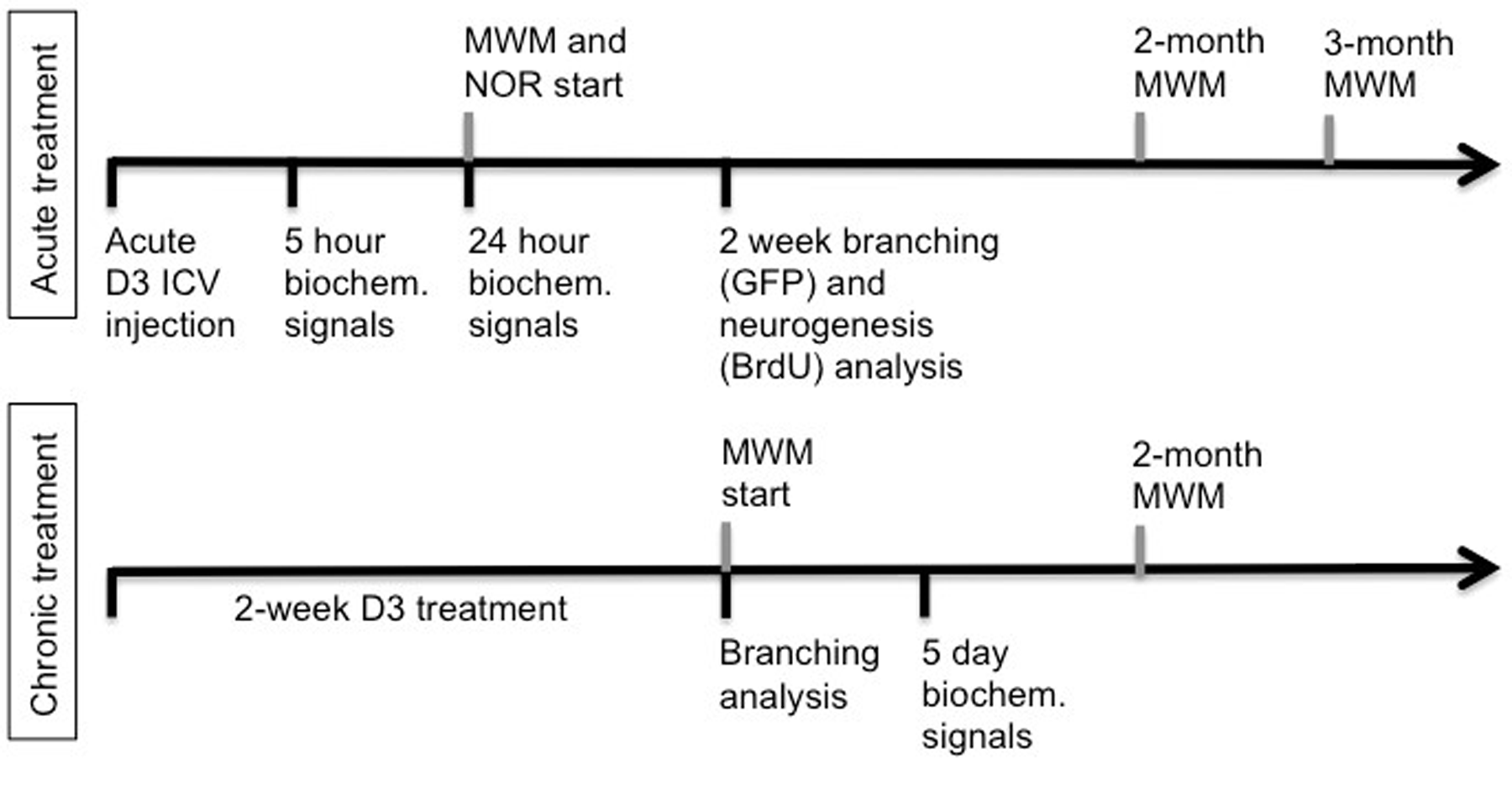

Supplement: S1 Fig — (TIF) [file pone.0218036.s001.tif]

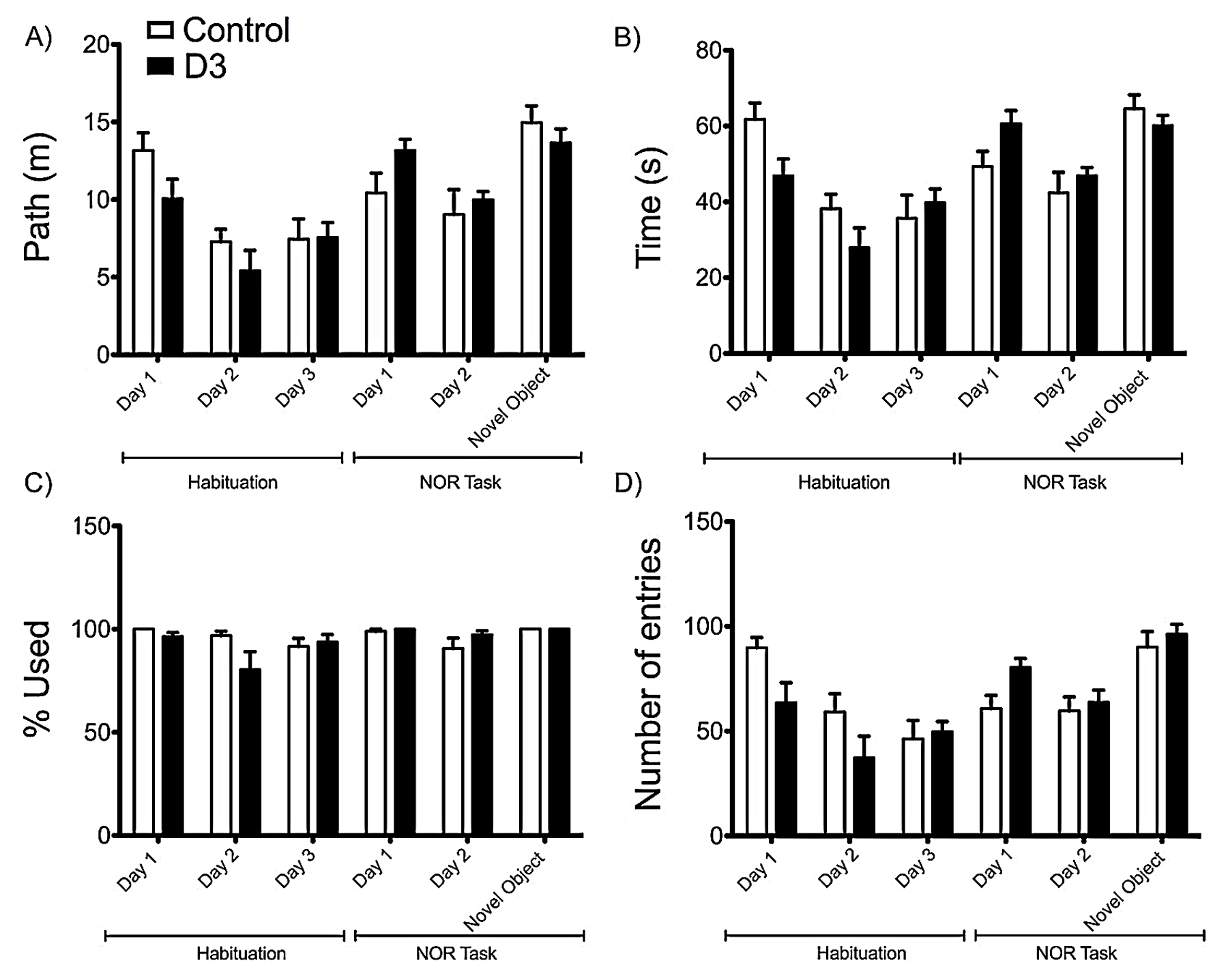

Supplement: S2 Fig — In all graphs, the first three days correspond to habituation, the following two days to familiarization (identical objects) and finally on the fifth day the novel object trial. A) The distance travelled by the mice was recorded in meters (p = 0.7). B) The time spent moving was recorded in seconds (out of a total of 300 s, p = 0.4). C) The percentage of the field explored by the mice (p = 0.9). D) The number of entries to the different quadrants in the field (p = 0.7). No significant differences were observed in any of the measurements (Controls n = 6, D3 n = 7; one repeat). Before the novel object recognition (NOR) test, mice were allowed to habituate to the testing environment. There were no differences between the controls and the D3-treated mice in the distance (path) traveled, the time spent exploring, the percentage of the space the mouse covered or the amount of times the mouse entered the different sections of the field. The same measurements were performed during object exploration and again we observed no differences between the two experimental groups. Both groups increased exploration when exposed to the objects for the first time and when exposed to the novel object (Controls n = 6, D3 n = 7). (TIF) [file pone.0218036.s002.tif]

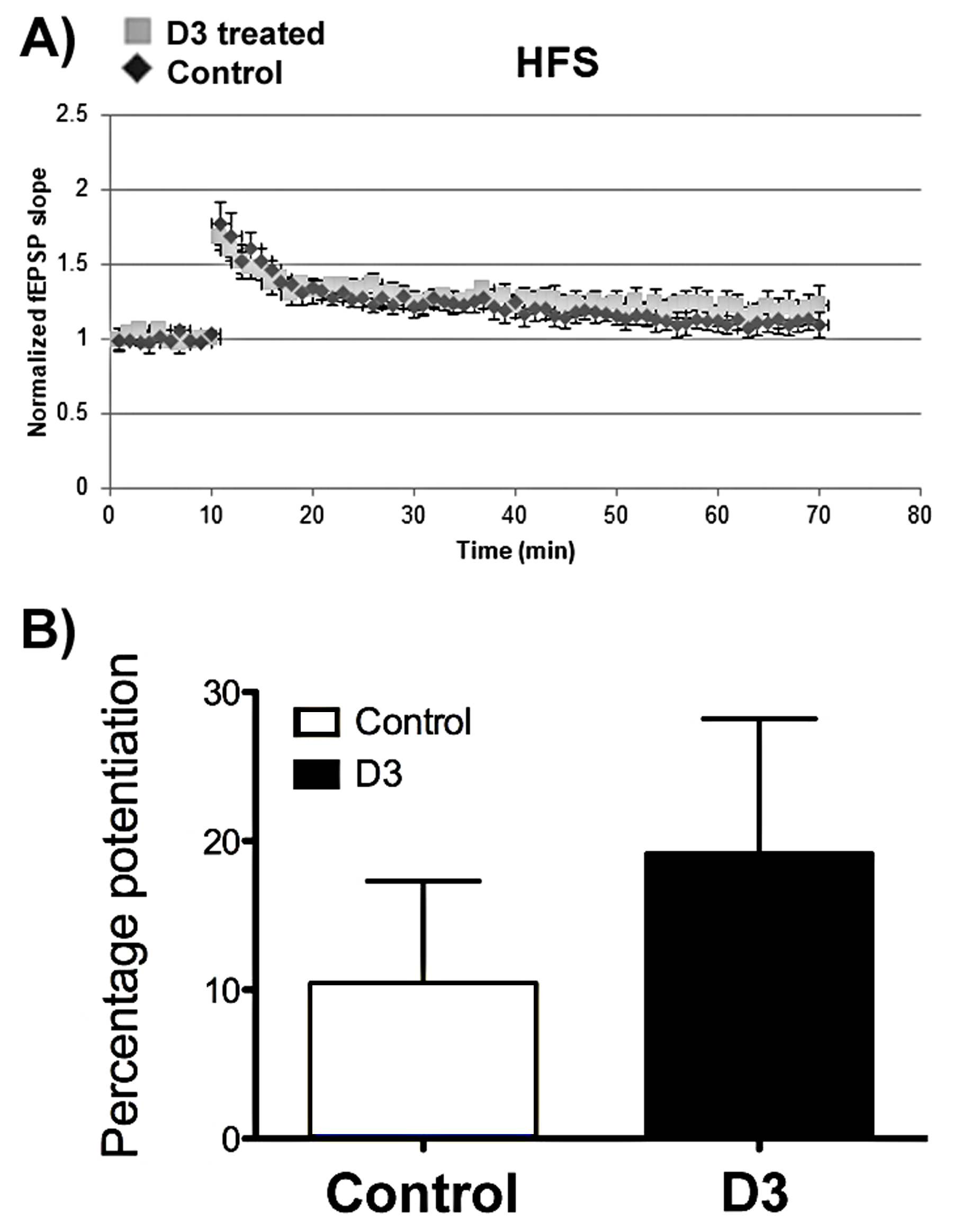

Supplement: S3 Fig — (A) Electrophysiological recordings were performed in mice 6 days after acute ICV injection with D3 or vehicle. (B) No significant differences were observed in percentage potentiation during the last 10 minutes, at 60 minutes after tetanus, a parameter corresponding to LTP (Controls n = 7 recordings, D3 n = 6 recordings; one repeat). To test whether D3 conveyed changes in LTP and baseline connectivity after acute ICV injection, electrophysiological analysis was performed. No differences were observed in LTP between D3 and vehicle-treated wild type mice, 6 days after acute ICV injections. No differences were observed in the input/output analysis either (data not shown). (TIF) [file pone.0218036.s003.tif]

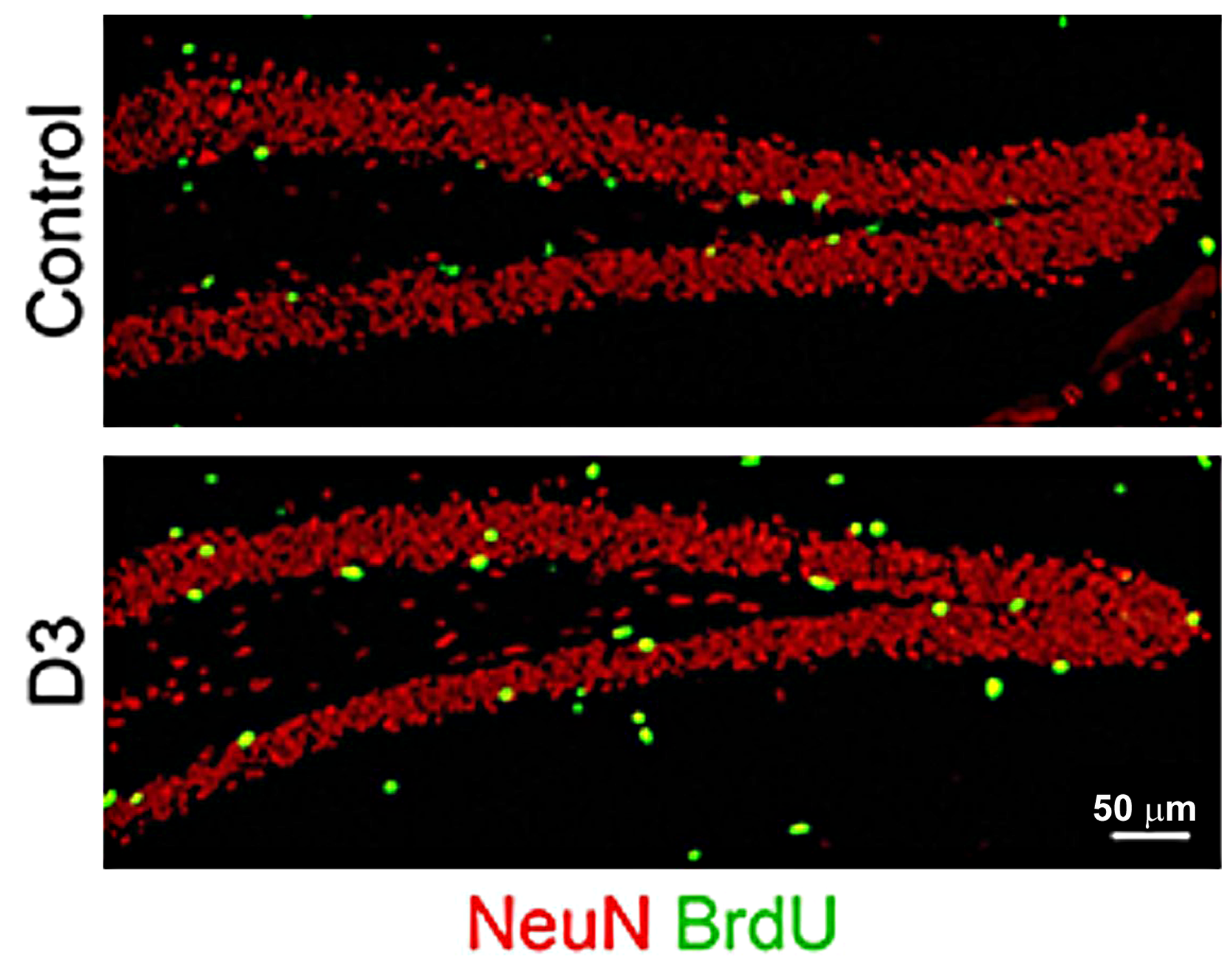

Supplement: S4 Fig — Mice were treated with aCSF or D3 (40 μg) ICV, simultaneously with BrdU PO. Immunofluorescence was performed for BrdU and NeuN. The amount of BrdU positive neurons was quantified in the subgranular zone of the dentate gyrus. No significant differences were observed between the controls (n = 2) and the D3-treated (n = 2) mice (p = 0.16). Conceivably, the decreases in dendrite branching in the CA1 region detected after D3-treatment may be caused by or coincide with defects in neurogenesis. Mice received chronic D3 ICV for 2 weeks, as well as, during the same period, BrdU in the water to label dividing cells. A non-statistically significant decrease in BrdU positive neurons was observed in the D3-treated mice (201±34 BrdU-positive neurons, n = 2) compared to controls (295±27 BrdU-positive neurons, n = 2) in the subgranular zone of the dentate gyrus of the hippocampus (unpaired 2-tailed t-test, p = 0.16, df = 2; n = 4 mice, approximately 30 sections per mouse, one repeat). The decrease in dendrite branching of the basal dendrites of neurons in the CA1 region was therefore independent of detectable effects on neurogenesis. BrdU Labeling in vivo, and Analysis. To study neurogenesis in vivo, BrdU was delivered at a concentration of 1 mg/mL in 1% glucose in drinking water. Mice were housed individually with individual water bottles containing BrdU, and the water consumed was constant between groups. BrdU was administered for the 2 weeks that the mice were administered chronically with D3 or aCSF. The mice were then perfused and their brains were fixed (4% PFA), processed for OCT, and cryo-sectioned into 12 μm thick slices (LEICA (Concord, Canada) 3050s cryostat). BrdU was exposed by submerging slides in 1N HCl at 45°C for 30 minutes. Cell membranes were permeabilized with 0.4% Triton X-100 PBS. Tissues were blocked with 5% NGS/3% BSA for 1 hour at room temperature. Slides were incubated with BrdU antibody 1:300 (Abcam ab6326) and NeuN 1:500 (Millipore mab-N78) in 0.2% Trito [file pone.0218036.s004.tif]

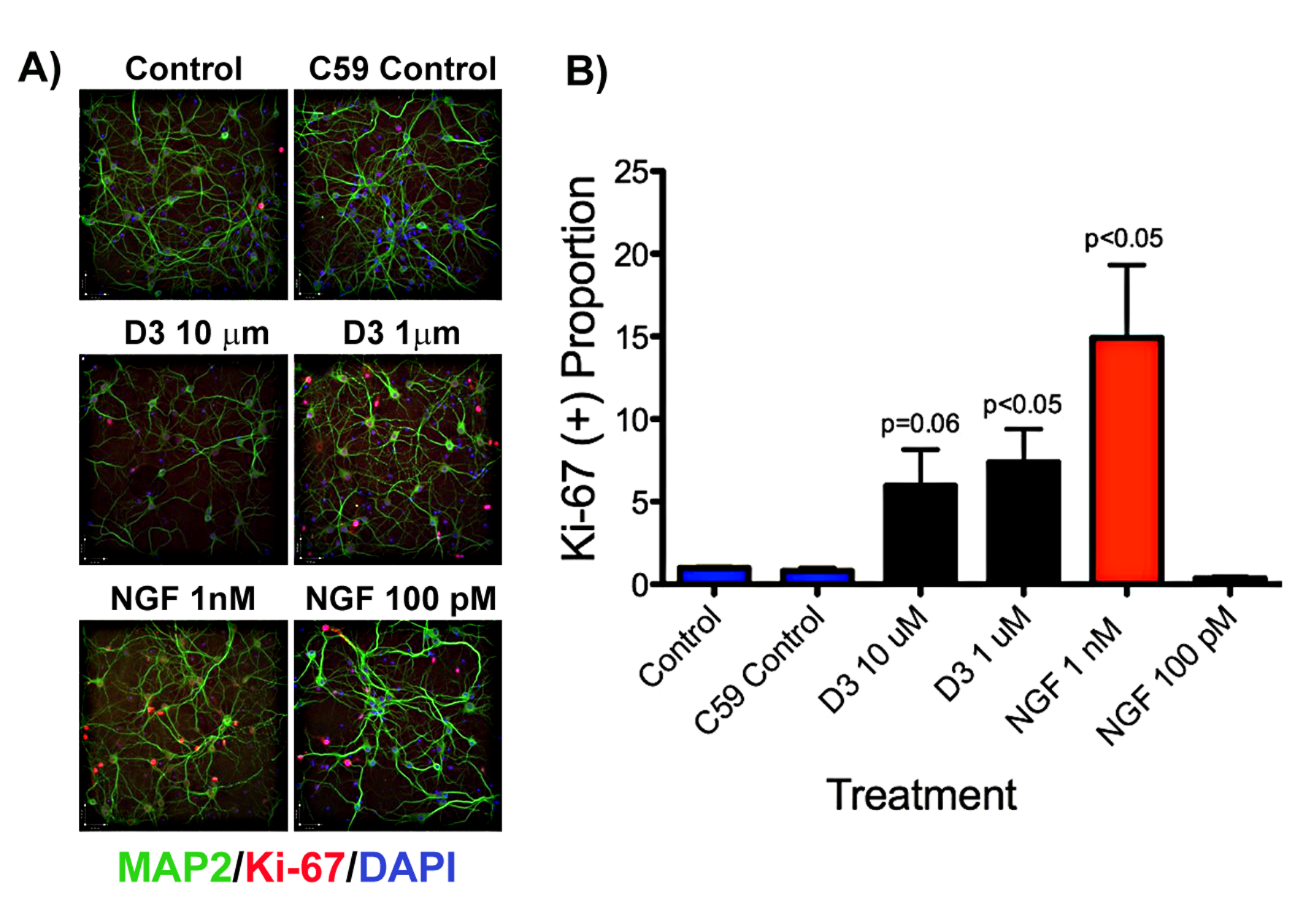

Supplement: S5 Fig — A) Primary embryonic hippocampal cultures were treated with supplemented Neurobasal medium (Control), and the following compounds in the same medium: C59 (an inert compound similar to D3), D3 at two different concentrations: 10 μM and 1 μM, and NGF at two different concentrations: 1 nM and 100 pM. Immunofluorescence was performed for Ki-67 (red) and MAP2 (green). B) Quantification of the proportion of Ki-67 positive neurons showed a significant increase in neurogenesis with D3 at both concentrations (p = 0.06 and p<0.05) and with NGF at 1 nM (p<0.05), two independent repeats. (TIF) [file pone.0218036.s005.tif]
